# A cooperative strategy for parameter estimation in large scale systems biology models

**DOI:** 10.1186/1752-0509-6-75

**Published:** 2012-06-22

**Authors:** Alejandro F Villaverde, Jose A Egea, Julio R Banga

**Affiliations:** 1 Bioprocess Engineering Group, IIM-CSIC, Eduardo Cabello 6, Vigo 36208Spain; 2 Department of Applied Mathematics and Statistics, Universidad Politécnica de Cartagena, Avenida Dr. Fleming s/n, 30202, Cartagena, Spain

## Abstract

**Background:**

Mathematical models play a key role in systems biology: they summarize the currently available knowledge in a way that allows to make experimentally verifiable predictions. Model calibration consists of finding the parameters that give the best fit to a set of experimental data, which entails minimizing a cost function that measures the goodness of this fit. Most mathematical models in systems biology present three characteristics which make this problem very difficult to solve: they are highly non-linear, they have a large number of parameters to be estimated, and the information content of the available experimental data is frequently scarce. Hence, there is a need for global optimization methods capable of solving this problem efficiently.

**Results:**

A new approach for parameter estimation of large scale models, called Cooperative Enhanced Scatter Search (CeSS), is presented. Its key feature is the cooperation between different programs (“threads”) that run in parallel in different processors. Each thread implements a state of the art metaheuristic, the enhanced Scatter Search algorithm (eSS). Cooperation, meaning information sharing between threads, modifies the systemic properties of the algorithm and allows to speed up performance. Two parameter estimation problems involving models related with the central carbon metabolism of *E. coli* which include different regulatory levels (metabolic and transcriptional) are used as case studies. The performance and capabilities of the method are also evaluated using benchmark problems of large-scale global optimization, with excellent results.

**Conclusions:**

The cooperative CeSS strategy is a general purpose technique that can be applied to any model calibration problem. Its capability has been demonstrated by calibrating two large-scale models of different characteristics, improving the performance of previously existing methods in both cases. The cooperative metaheuristic presented here can be easily extended to incorporate other global and local search solvers and specific structural information for particular classes of problems.

## Background

The aim of systems biology is to understand the organization of complex biological systems by combining experimental data with mathematical modeling and advanced computational techniques. Mathematical models play a key role, since–among other functions–they summarize the currently available knowledge in a way that allows to make experimentally verifiable predictions. Due to the increasing amount of “omic” data available from high throughput techniques, there is a need for large-scale model building methods.

Model building is a complex task that usually follows an iterative process [1-7]. It begins with the definition of the purpose of the model, this is, with the determination of the questions that the model should be able to answer. This conditions the modeling framework and the information that must be included in the model. The next step is to propose a mathematical structure with the necessary level of detail, which will in general include a number of unknown, non-measurable parameters. An estimation of these parameters is needed in order to obtain quantitative predictions; this is the next step, which is commonly known as parameter estimation or identification, data fitting, or model calibration [1,4-6]. The final step is model validation, which entails testing the model with new data; if this reveals modeling errors, the process should be iteratively repeated.

In recent years a number of approaches to large-scale kinetic modelling have been presented. Jamshidi and Palsson [8] presented a procedure to construct dynamic network models in a scalable way using metabolomic data mapped onto existing stoichiometric models, thus incorporating kinetics and regulation into those stoichiometric models. Liebermeister and Klipp [9,10] introduced a simplified, general rate law named convenience kinetics. It can be derived from a random-order enzyme mechanism, and it may be used to obtain a dynamical model from a biochemical network. The resulting model has plausible biological properties. More recently, the same authors proposed five modular rate laws [11], characterized by different formulae of their denominators, as a way of parameterizing metabolic network models. Tran et al [12] advocated the use of an ensemble of dynamic models. The models in the ensemble share the same mechanistic framework, span the space of all kinetics allowable by thermodynamics, and reach the same steady state. The size of the ensemble is reduced by acquiring data, converging to a smaller and more predictive set of models, which are able to describe relevant phenotypes upon enzyme perturbations. The well-known flux balance analysis method (FBA) has been widely used for large-scale analysis of metabolic networks. In [13] it was extended in order to incorporate transcriptional regulation (regulatory FBA, or rFBA); further developments included also signal transduction networks (integrated FBA or iFBA [14], and integrated dynamic FBA or idFBA [15]). Another integration of FBA with kinetic information was carried out by Smallbone et al. In [16] they presented a method for building a parameterized genome-scale kinetic model of a metabolic network, based solely on the knowledge of reaction stoichiometries. Fluxes were estimated by flux balance analysis and allowed to vary dynamically according to linlog kinetics. This method was applied [17] to a model of *S. cerevisiae* comprising 956 metabolic reactions and 820 metabolites. Kotte et al [18] modeled the coupling of enzymatic and transcriptional regulation of *E. coli*’s central metabolism using differential equations. The resulting medium-scale model is able to explain, from the interplay of known interactions, the adjustments of metabolic operation between glycolytic and gluconeogenic carbon sources. Another interesting contribution to the problem of building large-scale mathematical models of biological systems can be found in [19]. It extends an ordinary differential equation (ODE) model of ErbB signaling pathways to include 28 proteins, 499 ODEs, 828 reactions, and 229 parameters. The parameter space size is reduced to 75, following an initial sensitivity analysis based on estimates of nominal parameter values. Parameter estimation is then carried out by means of extensive computations using an stochastic method (simulated annealing).

The parameter estimation problem is an element of key importance in these modeling strategies. The aim is to find the parameters that give the best (optimal) fit to a set of experimental data, which entails minimizing (optimizing) a cost function that measures the goodness of this fit. Thus, calibration of dynamic models can be considered as one of the applications of mathematical optimization in computational systems biology [20]. Parameter estimation is usually formulated as a non-linear programming problem (NLP) subject to the dynamic and stationary constraints which define the behaviour of the system. Most mathematical models in systems biology present three characteristics which make this problem very difficult to solve: 

1. They are highly non-linear, which creates multimodality, so standard local methods (e.g. Levenberg-Marquardt or Gauss-Newton) converge to local solutions. This must be overcome with the use of global methods capable of finding the optimum in a rugged landscape.

2. There is large number of parameters to be estimated. This is an especially problematic issue, since the necessary computational effort increases very rapidly with the problem size. The increase may be exponential, thus preventing methods that work well for a limited number of parameters from being applied to realistically sized problems. This means that global deterministic methods, which guarantee finding the global optimum, fail to provide the solution in reasonable computing times. Hence, stochastic methods must be used instead.

3. The information content of the available experimental data is frequently scarce, which might cause an identifiability problem (i.e., different combinations of parameter values may produce similar model outputs). An example illustrating this point can be found in the aforementioned work of Chen et al [19], where the estimation of parameters yielded the result that the model is non-identifiable.

Due to these difficulties, no full and proper model calibration has been performed so far in the large-scale kinetic models referenced above. In many cases, a large subset of parameters were taken, where available, from previous models or databases, but this procedure, although practical for some aplications, is not equivalent to a proper calibration of the large-scale model. Our main objective in this paper it to present a novel metaheuristic which exploits cooperative parallelism in order to surmount the difficulties mentioned above. Parallel implementations of global optimization methods have been used recently in systems biology (see [21] and references therein). Here we have improved this concept by incorporating cooperation of individual search threads.

The novelty of our approach has the following two main pillars: 

• The use of an efficient global optimization method for solving large-scale parameter estimation problems, based on extensions of the scatter search metaheuristic [22-25].

• A parallel implementation of the algorithm incorporating a cooperative strategy, which means that there is an exchange of information between a set of individual optimization programs, or threads, running in parallel. As a result, this cooperation not only speeds up the algorithm, but also alters its systemic properties, thus yielding performance improvements more than proportional to the increase in computing power.

The capabilities of these novel methods are illustrated considering two case studies based on *E. coli*: 

• Model 1: a model of *E. coli*’s central carbon metabolism (CCM) [11].

• Model 2: a dynamic *E. coli* model that couples its central carbon metabolism with enzymatic and transcriptional regulation [18].

This paper is structured as follows: after the presentation of the problem statement, we discuss the need of global optimization methods, and then we present a cooperative parallel method which is able to handle the challenges arising from this class of problems. Results for two different case studies are then presented and discussed, finally arriving to a set of conclusions.

### Problem statement

Given a model of a nonlinear dynamic system and a set of experimental data, the parameter estimation problem consists of finding the vector of decision variables **p**(unknown model parameters) that minimizes a cost function that measures the goodness of the fit of the model predictions with respect to the data, subject to a number of constraints. The output state variables that are measured experimentally are called observables.

Mathematically, it is formulated as a nonlinear programming problem (NLP) with differential-algebraic constraints (DAEs), where the goal is to find **p** to minimize

(1) $$J=\overset{n_{\varepsilon }}{\underset{\varepsilon =1}{\sum }}\overset{n_{o}^{\varepsilon }}{\underset{o=1}{\sum }}\overset{n_{s}^{\varepsilon ,o}}{\underset{s=1}{\sum }}{\left(ym_{s}^{\varepsilon ,o}-y_{s}^{\varepsilon ,o}(p)\right)}^{T}W\left(ym_{s}^{\varepsilon ,o}-y_{s}^{\varepsilon ,o}(p)\right)$$

where *n*_*ε*_ is the number of experiments, $n_{o}^{\varepsilon }$ is the number of observables per experiment, and $n_{s}^{\varepsilon ,o}$ is the number of samples per observable per experiment. The measured data will be denoted as ${ym}_{s}^{\varepsilon ,o}$ and the corresponding model predictions will be denoted as $y_{s}^{\varepsilon ,o}$ (**p**). Finally, *W * is a scaling matrix used to balance the contributions of the observables (usually by scaling each temporal series with respect to its maximum value).

The minimization is subject to the following constraints:

(2) $$\dot{x}=f\left(x,p,t\right)$$

(3) $$x(t_{0})=x_{0}$$

(4) y=g(x,p,t)

(5) $$h_{\text{eq}}(x,y,p)=0$$

(6) $$h_{\text{in}}(x,y,p)\leq 0$$

(7) $$p^{L}\leq p\leq p^{U}$$

where *g* is the observation function, *x* is the vector of state variables with initial conditions *x*_0_, *f * is the set of differential and algebraic equality constraints describing the system dynamics (that is, the nonlinear process model), *h*_*eq *_and *h*_*in*_ are equality and inequality constraints that express additional requirements for the system performance, and **p**^*L*^ and **p**^*U*^ are lower and upper bounds for the parameter vector **p**.

As was mentioned in the Background section, in systems biology models this problem is often multimodal (nonconvex), due to the nonlinear and constrained nature of the system dynamics. Hence, standard local methods usually fail to obtain the global optimum. As an alternative, one may choose a multistart strategy, where a local method is used repeatedly, starting from a number of different initial guesses for the parameters. However, this approach is usually not efficient for realistic applications, and global optimization (GO) techniques, such as the ones presented in the next section, need to be used instead.

## Methods

To efficiently solve the calibration problem there is a need of global optimization methods whose performance does not deteriorate when the number of parameters to be estimated is very large (as it occurs with most methods). Global optimization methods can be divided into deterministic and stochastic. Deterministic global optimization methods guarantee that the solution is the global optimum, but the computational effort they require can make them unaffordable for large-scale problems. Stochastic global optimization methods, on the other hand, do not guarantee the global optimality of the solution, but they are frequently capable of finding excellent solutions in reasonable computation times. A particularly efficient class of stochastic global optimization methods are the so-called metaheuristic approaches, which combine mechanisms for exploring the search space and exploiting previously obtained knowledge. Here we propose a method, Cooperative enhanced Scatter Search (CeSS), based on extensions and cooperative parallelization of the scatter search metaheuristic [22-25].

### Global optimization via enhanced scatter search

Scatter search can be classified as an evolutionary optimization method which, like many other metaheuristics, arose in the context of integer optimization [26]. Several adaptations to continuous problems have been developed in recent years. The application of the method to parameter estimation in systems biology problems has provided excellent results [22].

Scatter search is a population-based algorithm which, compared with other methods like genetic algorithms, makes use of a low number of population members, called “Reference Set” (*RefSet*) in this context. Besides, scatter search includes the so-called “improvement method” which usually consists in a local search to speed-up the convergence to optimal solutions.

**Figure 1:**
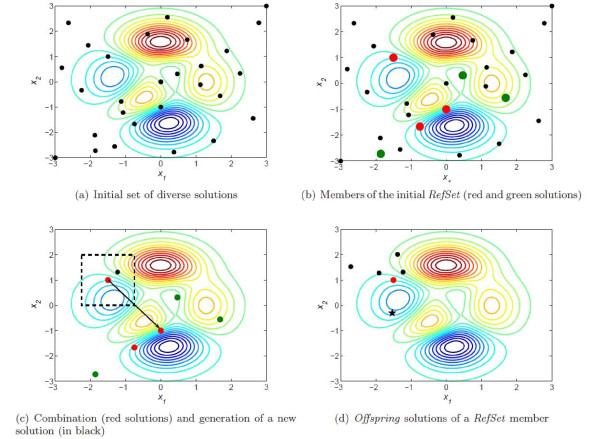
**eSS algorithm.** Enhanced scatter search scheme.

To illustrate how the method works, Figures 1.(a-d) represent the contour plots of an arbitrary objective function to be optimized (e.g., a least squares function in the case of parameter estimation), together with the solutions which constitute the *RefSet* and their evolution.

The method starts by creating an initial population of diverse solutions within the search space (Figure 1.a). From them, the best solutions in terms of quality are selected to create the initial *RefSet* (in red in Figure 1.b). The rest of solutions in the *RefSet* are chosen randomly (in green in Figure 1.b), although in some advanced implementations they may be chosen following a criterion of maximum diversity to cover a broader area of the search space. Once the *RefSet* solutions have been selected, the remaining diverse solutions generated in the first step are deleted and the so-called “subset generation method” starts. It consists in combining sets of *RefSet* solutions to create new ones. In our scheme, we combine every solution in the *RefSet* with the other *RefSet* by generating hyper-rectangles defined by their relative position and distance, and creating new solutions inside them. This step is illustrated in Figure 1.c. Each *RefSet* member will create one hyper-rectangle with every other *RefSet* member, thus generating a number of *b*−1 new solutions (being *b* the *RefSet* size) per each *RefSet* member. After this procedure, all the *RefSet* members have a set of *offspring* solutions around them covering different distances and directions (Figure 1.d). If the best *offspring* solution outperforms the parent solution (which is the case of Figure 1.d with the best *offspring* solution represented as a star), the replacement (or update) is carried out. Otherwise, the same *RefSet* member will stay in the population in the next iteration. Although the procedure is illustrated for just one *RefSet* member, the same scheme applies for every solution in the *RefSet*. After the *RefSet* update, the algorithm applied the “solution combination method” again, repeating the process until a stop criterion is met.

In this work, a novel extended implementation of the enhanced scatter search metaheuristic (eSS) [24,25], has been used. Its pseudocode is shown in Algorithm 1.

### Algorithm 1

Basic pseudocode of *eSS*

Set parameters: *dim_refset*, *local.n*2, *balance*, *ndiverse*

Initialize *n*_*stuck*_, *neval*

Create set of *ndiverse* solutions

Generate initial *RefSet* with *dim_refset* solutions with high quality and random elements

repeat

Sort *RefSet* by quality [*x*^1^,*x*^2^,…,*x*^*dim_refset*^]so that *f*(*x*^*i*^) ≤* f*(*x*^*j*^) where *i*,*j*∈[1,2,…,*dim_refset*] and *i *<* j*

**if** max $\left(\text{abs}\left(\frac{x^{i}-x^{j}}{x^{j}}\right)\right)\leq \varepsilon$ with *i *<* j ***then**

Replace *x*^*j *^by a random solution

end if

**for ***i *= 1 to *dim_refset ***do**

Combine *x*^*i *^with the rest of population members to generate a set of *dim_refset* new solutions,

**offspring**^*i*^

$x_{\text{off}}^{i}=$ best solution in **offspring**^*i*^

**if**$x_{\text{off}}^{i}$ outperforms *x*^*i *^**then**

Label *x*^*i*^

*x*_temp_ =* x*^*i *^

*improvement *= 1

*∧ *= 1

**while**$f(x_{\text{off}}^{i})<f(x_{\text{temp}})$**do**

Create a new solution, *x*_new_, in the hyper-rectangle defined by $\left[x_{\text{off}}^{i}-\frac{x_{\text{temp}}-x_{\text{off}}^{i}}{\wedge },x_{\text{off}}^{i}\right]$

$x_{\text{temp}}=x_{\text{off}}^{i}$

$x_{\text{off}}^{i}=x_{\text{new}}$

*improvement *=* improvement* + 1

**if*** improvement *= 2** then**

*∧ *=* ∧*/2

*improvement *= 0

end if

end while

end if

end for

**offspring**= $\left(\begin{array}{c} {\text{offspring}}^{1}\\ {\text{offspring}}^{2}\\ \ldots \\ {\text{offspring}}^{n} \end{array}\right)$ with *n *=* dim_refset*

**if ***neval *≥* local.n*2** and** at least one local solution has been found **then**

Sort **offspring** solutions by quality [*x*_1,1_,*x*_2,1_,…,*x*_*m*,1_] where *m *=* n*(*n*−1) and *f*(*x*_*i*,1_) ≤* f*(*x*_*j*,1_) where *i*,*j*∈[1,2,…,*m*] and *i *<* j*

Compute the minimum distance between each element *i *∈ [1,2,…,*m*] in **offspring** and all the local optima found so far, *d*(*x*_*i*_)

Sort **offspring** solutions by diversity [*x*_1,2_,*x*_2,2_,…,*x*_*m*,2_] with *d*(*x*_*i*,2_) ≥* d*(*x*_*j*,2_) where *i*,*j*∈[1,2,…,*m*] and *i *<* j*

Choose *z* as the **offspring** member balancing quality and diversity according to *balance*

Perform a local search from *z* to obtain *z*^∗^

**if ***z*^∗^ is a new local optimum **then**

Update list of found local optima adding *z*^∗^

end if

**if ***f*(*z*^∗^) <* fbest ***then**

Update *xbest*, *fbest*

end if

*neval *= 0

end if

*neval *=* neval* + function evaluations of current iteration

Replace labeled population members by their corresponding $x_{\text{off}}^{i}$ and reset their corresponding *n*_*stuck*_(*i*)

Add one unit to the corresponding *n*_*stuck*_(*j*) of the not labeled population members

**if** any of the *n*_*stuck *_values >* nchange ***then**

Replace those population members by random solutions and reset their *n*_*stuck *_values

end if

**until** stopping criterion is met

Apply final local search over *xbest* and update it

The innovative mechanisms implemented in different parts of the method as well as the appropriate local search selection, make the algorithm specially suitable for solving large-scale optimization problems. These mechanisms, which address the most important question in global optimization (i.e., the balance between intensificacion, or local search, and diversification, or global search) are depicted in the following points: 

• Population-based algorithms with a large population size can become inefficient due to the large amount of function evaluations they perform in each iteration. On the other hand, if the population size is too small, the algorithm may converge prematurely to suboptimal solutions. The enhanced scatter search method uses a small population size, even for large-scale problems, but allowing more search directions than in classical scatter search designs thanks to the hyper-rectangle-based combination scheme. This does not increase the number of evaluations per iteration while preserving the diversity in the search.

• Another key aspect in population-based algorithms is the population update scheme. (*μ* + *λ*) strategies [27] (i.e., members of the following population will be selected among the set of solutions including old population members and new *offspring* solutions) may tend to prematurely stagnate the population in suboptimal solutions. (*μλ*) strategies (i.e., members of the following population will be selected among the set of solutions including only new *offspring* solutions) may need a high number of function evaluations to converge. On the other hand a (1 + 1) strategy applied to every population member as the one depicted in Figures 1.(a-d) can take the advantages of both mechanisms. This is the strategy used in eSS and, again, provides a good balance between intensification (i.e., local search) and diversification (i.e., global search).

• The specific intensification mechanisms are crucial in large scale optimization in order to reduce the computational times as much as possible. eSS implements an intensification mechanism already in the global phase, which exploits the promising directions defined by a pair of solutions in the *RefSet*. Besides, the use of a local search method to accelerate the convergence is mandatory in this type of applications. Gradient-based methods are the most widely used local methods. However, for dynamic problems they might be inefficient due to some characteristics of this type of problems (see [28] for more details). Furthermore, the calculation of the numerical sensitivities carried out by these methods may become unaffordable when the problem dimension is too high. A heuristic local search method seems to be the most convenient choice when dealing with large-scale problems because, even if the local convergence might not be ensured, avoiding the calculation of sensitivities or search directions may save a large amount of calculation time. For this reason, and after extensive initial tests, we have selected a heuristic method known as “Dynamic Hill Climbing” [29], available in the eSS framework. This method provided the best results for the problems tested.

• Even if intensification strategies should be favoured in large-scale optimization due to the limited computational time usually available in real applications, diversification strategies should not be neglected because stagnation of the search may appear even in early stages of the process. In this regard, eSS implements diversification strategies which make use of memory to infer whether a solution is stagnated in a local optimum or if it is too close to previously found solutions. When these solutions are identified, they are automatically replaced to avoid spending computational effort searching in areas already explored.

### Cooperative optimization

A common way of reducing the computational cost of a given algorithm is to parallelize it. This entails performing several of its calculations simultaneously in different processors. Except for some special cases, which are usually called “embarrasingly parallel”, it is not trivial to separate the problem into parallel tasks. Here we are interested in the parallelization of a metaheuristic optimization algorithm, the enhanced scatter search method described in the previous section.

Before we describe the proposed methodology, we clarify the use of a few words in order to avoid confusion. We refer to a context where there are a number of *processors* available for computing, which can work simultaneously (in *parallel*). All of the processors are assumed to be physically identical, that is, they have the same hardware characteristics. A *program* running in a processor is called *thread*; both terms are interchangeable. In our methodology we use a *master* processor, with the task of coordinating the remaining processors (*slaves*). All of the slave processors implement the same program, which means they perform similar calculations, using different data sets and different settings.

There are not many examples of research in the area of parallelization of metaheuristics exploiting cooperation in the way we present here. In [30] three different parallelizations of the scatter search were proposed and compared. In the first one, each local search was parallelized. In the second one, several local searches were carried out in parallel. In these two cases it was possible to reduce the computational time. A third option was to run the algorithm for different populations in parallel, but without any communication between threads.

More recently [31] a more ambitious approach was presented, where different kinds of metaheuristics were combined in order to profit from their complementary advantages. Information was shared between algorithms and it was dynamically tuned, so the most succesful algorithms were allowed to share more information. For a detailed review of the state of the art in parallelization of metaheuristics, see [32].

Sharing information can be seen as a way of *cooperating*. Such cooperation produces more than just speed-up: it can change the systemic properties of an algorithm and therefore its macroscopic behaviour. This was acknowledged in [33], where four parameters that may affect this behaviour were identified: (i) information gathered and made available for sharing; (ii) identification of programs that share information; (iii) the frequency of information sharing among the sequential programs; and (iv) the number of concurrent programs.

In this section we explore the possibilities of cooperation in order to speed up the convergence of the enhanced Scatter Search methodology. We have developed a strategy that is in some way intermediate between the two aforementioned approaches of [30] and [31]. The idea is to run, in parallel, several implementations or *threads* of the optimization algorithm–which may have different settings and/or random initializations–and exchange information between them. According to the classification proposed in [33], this arrangement is characterized as follows: 

1. Information available for sharing: the best solution found and, optionally, the reference set (*RefSet*), which contains information about the diversity of solutions.

2. Threads that share information: all of them.

3. Frequency of information sharing: the threads exchange information at a fixed interval *τ*.

4. Number of concurrent programs: *η*.

Each of the *η*threads has a fixed degree of “aggressiveness”. “Conservative” threads make emphasis on diversification (global search) and are used for increasing the probabilities of finding a feasible solution, even if the parameter space is “rugged” or weakly structured. “Aggressive” threads make emphasis on intensification (local search) and speed up the calculations in “smoother” areas. Please note that both conservative and agressive threads should be able to locate the globally optimal solution of the class of problems considered here, but their relative efficiency will vary depending on the region of the parameter space where the individual threads are operating. In this way, we achieve a synergistic gain. Communication, which takes place at fixed time intervals, enables each thread to benefit from the knowledge gathered by the others. This knowledge may include not only information about the best solution found so far, but also about the sets of diverse parameter vectors that may be worth trying for improving the solution. Thus this strategy has several degrees of freedom that have to be fixed: the time between communication (*τ*), the number of threads (*η*), and the strategy adopted by each thread (*Ψ*). These adjustments should be chosen carefully depending on the particular problem we want to solve. In the following lines we give some guidelines for doing this.

#### Time between information sharing, *τ*

On the one hand, the time between information sharing must be large enough to allow each of the threads to exploit the algorithm’s capabilities. This includes the initial generation of diverse solutions and their evaluation, followed by several iterations which usually include local searches. The necessary time depends on the thread’s settings, which determine, among other things, the number of diverse solutions that are generated. It also depends on the time required for an evaluation of the objective function. On the other hand, if the time is too large, cooperation will happen only sporadically, and its efficiency will be reduced. Hence the appropriate value should be chosen from a compromise, taking into account the time required by a single thread to complete a predefined number of function evaluations, and the problem dimension (i.e. the number of parameters, *n*_*par*_). We define a tuning parameter *τ*as

(8) $$\tau =\text{lo}g_{10}\left(\frac{n_{\text{eval}}}{n_{\text{par}}}\right)$$

where *n*_*eval *_is the maximum number of function evaluations allowed between cooperation instants. Therefore, *τ*, as defined above, is a dimensionless and machine-independent parameter that sets the duration of the intervals between information sharing. The logarithm is used for the convenience of avoiding very large numbers. For all the problems considered in this study, we have empirically found that a value in the interval 2.5 <* τ *< 3.5, with a default value of 2.5, results in a good trade-off between efficiency and robustness of the cooperative strategy.

#### Number of concurrent threads, *η*

As *η *increases, the expected speed up increases too. However, this increase cannot grow in a linear way for an arbitrarily large number of threads, due to a number of reasons. One reason is that, as the number of threads increases, so does the communication overhead. More importantly, the risk of overlapping - that is, carrying out similar searches in different threads - increases too due to the relatively small size of the RefSet. Hence, when the number of threads is very large, the resulting speed up is smaller than the increase in computational effort. For the problems considered here, we have empirically found that an *η *= 10 led to good results and thus can be recommended as a default value.

#### Settings (strategy) for each thread, *Ψ*

For cooperation to be efficient, every thread running in parallel must perform different calculations. Due to the random nature of metaheuristic algorithms, this can be achieved simply by choosing different random initializations, or “seeds”, for each thread. This results first in different sets of initial guesses, and subsequently in the exploration of different points of the search space. However, this is not the only difference that may be made between threads: we can also select a different behaviour (more or less aggressive) for each of the threads. An “aggressive” thread performs frequent local searches, trying to refine the solution very quickly, and keeps a small reference set of solutions. This means that it will perform well in problems with parameter spaces that have a smooth shape. “Conservative” threads, on the other hand, have a large reference set and perform local searches only sporadically. This means that they spend more time combining parameter vectors and exploring the different regions of the parameter space. Thus, they are more robust, and hence appropriate, for problems with rugged parameter spaces. Since the exact nature of the problem to consider is always unknown, it is advisable to choose a range of settings that yields conservative, aggressive, and intermediate threads. For the eSS algorithm this can be obtained by tuning the following settings: 

• Number of elements in the Reference Set (*dim_refset*).

• Minimum number of iterations of the eSS algorithm between two local searches (*local.n2*).

• Balance between intensification and diversification in the selection of initial points for the local searches (*balance*).

• Number of diverse solutions initially generated (*ndiverse*).

All these settings have qualitatively the same influence on the algorithm’s behaviour: if they are large, they lead to conservative threads; if they are small, to aggressive threads. The four parameters that define the degree of aggressiveness of each of the *η* threads can be stored in an array *Ψ*_*η*×4_. By default, we recommend to use a broad spectrum of aggressiveness using the default values 0.5·*n*_*par *_<* dim*_*refset *< 20·*n*_*par*_; 0 <* local.n*2 < 100; 0 <* balance *< 0.5; and 5·*n*_*par *_<* ndiverse *< 20·*n*_*par*_.

**Figure 2:**
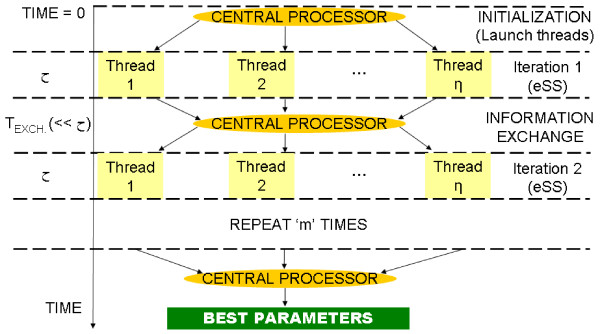
**Cooperative algorithm.** Scheme of the cooperative algorithm; *η *threads exchange information at fixed intervals with periodicity *τ*, as defined in eq. (8).

Figure 2 shows the cooperative loop, and Algorithm 2 summarizes its pseudo-code. The text marked as SLAVE, PROCESSOR *j* corresponds to the code implemented in parallel by each of the slave processors; the rest of the computations are carried out by the master processor.

### Algorithm 2

Basic pseudocode of the cooperative strategy

MASTER, PROCESSOR 0: Initialization

Set parameters that are common for all threads: *τ*, *η*, *n*_*iters*_

Set array of aggressiveness parameters: *Ψ*_*η*×4_ = {*dim_refset*_*η*×1_,*ndiverse*_*η*×1_,*local.n2*_*η*×1_,*balance*_*η*×1_}

Initialize global reference set array: *Global*_*ref *_= []

**for ***i *= 1 to *n*_*iters *_**do**

**for ***j *= 1 to *η ***do**

SLAVE, PROCESSOR *j* (concurrent processing): run optimization according to Algorithm 1

(Thread *j* uses aggressiveness settings vector *Ψ*_{*j*,:}_ and initial points *Global*_*ref*_)

end for

**if** CPUtime ≥* τ ***then**

**for ***j *= 1 to *η ***do**

Read results of thread *j*

**if ***ref*_*j *_∉* Global*_*ref *_**then**

*Global*_*ref *_= [*Global*_*ref*_,*ref*_*j*_]

end if

end for

end if

end for

Final solution = best solution in *Global*_*ref*_

## Results and discussion

As a preliminary validation, we first applied CeSS to a problem taken from the competition on Large Scale Global Optimization of the 2012 IEEE World Congress on Computational Intelligence [34]. Testing the performance of our method with this problem has two advantages: first, the benchmark is well known in the large-scale global optimization community, which facilitates the critical examination of the results; and second, the objective function selected from the benchmark has a smaller computational cost than the ones from our models (despite having one thousand parameters), which allows to carry out more extensive tests in less time. The results are shown in the supplementary material, see Additional file 1. From them it is concluded that eSS performs well with the benchmark problem, and that its performance is considerably improved by using its cooperative version, CeSS. This confirms the validity of the proposed approach for large-scale global optimization problems.

Next, we applied CeSS to the two *E. coli* models mentioned in the Background section. *Model 1* describes its central carbon metabolism (CCM), modeled with the common modular rate law [11]. *Model 2* couples its CCM with enzymatic and transcriptional regulation [18]. Due to its dynamic character, the simulation of Model 2 has a larger computational cost: in our computers (Xeon Quad-core processor, 2.50 GHz, 3 GB/core), one evaluation of the objective function lasts tenths of seconds for Model 1 and several seconds for Model 2. The models characteristics are summarized in Table 1. The CeSS method described above was implemented and executed in Matlab. Results are discussed in the following subsections.

**Table 1:** Overview of model features

	**Model 1**	**Model 2**
Levels	Metabolic	Metabolic, transcriptional
Parameters	918	178
States	115	47
Observables	115	47
Measures	1150	7614
Static / Dynamic	Static	Dynamic

*Model 1*: states are initial concentrations of internal metabolites and initial values of metabolic fluxes. Data corresponds to 10 different simulated experiments. For each experiment, the enzyme levels and external metabolite levels are randomly chosen. Then the metabolic fluxes and internal metabolite levels can be obtained solving for the steady state. The optimization goal is to minimise the residuals for the fluxes and internal metabolite levels, which are assumed to be measurable. More information about the model is included in the Additional file 2.

*Model 2*: states are the species concentrations; data corresponds to extended diauxic shift scenario consisting of 3 consecutive environments: first the carbon source is glucose, then acetate, and finally a mixture of both. The scenario is described in the simulation files included as supplementary material in [18]. For simulation purposes it is assumed that all of the states are measured. More information about the model is included in the Additional file 3.

### Model 1: *E. coli* CCM

First we consider a model of *E. coli*’s central carbon metabolism (CCM) with 66 reactions and 74 metabolites, 49 internal and 25 external (Wolfram Libermeister, personal communication). Reactions and metabolites are taken from the Kyoto Encyclopedia of Genes and Genomes database ID [35]; see Additional file 2 for a list of their names and KEGG IDs. Reaction kinetics are modeled with the common modular (CM) rate law proposed in [11], which is a generalized form of the reversible Michaelis-Menten kinetic that applies to any reaction stoichiometry. For a non-regulated reaction A + B ↔ 2C with concentrations *a*, *b* and *c*, the rate in mol/s reads:

(9) $$v=u\frac{k^{+}\frac{a}{k_{A}^{M}}\frac{b}{k_{B}^{M}}-k^{-}{\left(\frac{c}{k_{C}^{M}}\right)}^{2}}{\left(1+\frac{a}{k_{A}^{M}}\right)\left(1+\frac{b}{k_{B}^{M}}\right)+{\left(1+\frac{c}{k_{C}^{M}}\right)}^{2}-1}$$

where *u* is an enzyme level (mmol), $k_{A}^{M}$, $k_{B}^{M}$ and $k_{C}^{M}$ are reactant constants (mM), and *k*^±^ are turnover rates (s^−1^). The CM rate law resembles the convenience kinetics, with a slight difference for molecularities *m*^±^ ≠ 1 (in convenience kinetics, the denominator term for product C would read $1+\frac{c}{k_{C}^{M}}+{\frac{c}{k_{C}^{M}}}^{2}$).

The resulting model has 918 parameters: internal metabolites concentrations; activation, inhibition, and Michaelis-Menten constants; and velocity and equilibrium constants. Their values are given in the supplementary material, see Additional file 2. They are estimated by least squares minimization of the difference between the model predictions and artificially generated data regarding internal metabolic concentrations (49) and fluxes (66), yielding a state vector with 115 elements.

**Figure 3:**
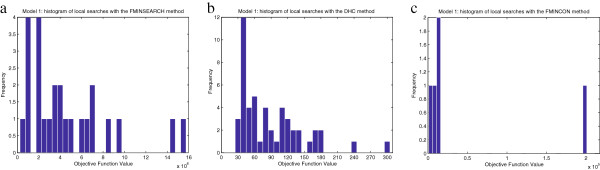
**Model 1 - Histograms of multistart local searches.** Objective function values achieved by local searches starting from different points with the FMINSEARCH **(Figure**3**.a)**, DHC **(Figure**3**.b)**, and FMINCON **(Figure**3**.c)** methods. Calculations were carried out in a single processor and the computation time was 10 days for each method. The best values obtained were: 5.367·10^5^(FMINSEARCH), 2.938·10^1^(DHC), and 1.459·10^3^(FMINCON).

Multistart procedures of local methods are carried out in order to estimate the nonconvexity of the problem and compare the results with those obtained with global optimization. The upper and lower bounds allowed for the parameters are, respectively, 10 times larger and 10 times smaller than the nominal values, which are shown in the Additional file 2. Several local methods are tested: Direct Hill Climbing (DHC), and the MathWorks functions *fminsearch* (for unconstrained minimization) and *fmincon* (for constrained minimization). A common computation time of ten days is imposed to every one of them. Histograms of the solutions are shown in Figure 3; the best value of the objective function found is *f*_*best *_= 2.94·10^1^, which is achieved with the DHC method. It can be noticed that for the same computation time, some algorithms perform more local searches than others. For example, with *fmincon* the calculation of the gradient is very costly due to the large number of decision variables, while DHC avoids these calculations and is thus capable of completing more local searches in the same time.

Suspecting that a more sophisticated method may be able to find a better solution, we test our enhanced Scatter Search (eSS) algorithm. Since the multistart calculations were carried out in a single processor, with a total running time of 10 days, it is fair to compare the results with those obtained by 10 processors in one day. We thus select 10 different settings for the eSS algorithm and launch the corresponding 10 optimizations. Since eSS provides the possibility of launching local searches, we select the local method that yields best results with the multistart procedure, DHC. After one day, the best objective function value achieved is *f*_*best *_= 7.86, which represents a reduction of 73% with respect to what was obtained with a multistart of local searches.

**Figure 4:**
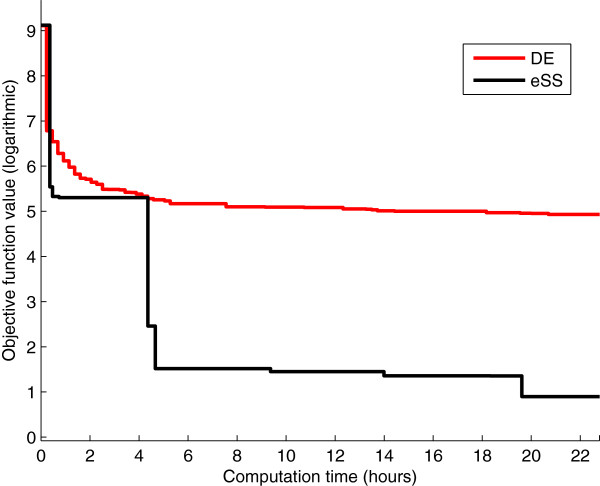
**Model 1 - Differential Evolution vs. non-cooperative enhanced Scatter Search.** Convergence curves of the DE and eSS methods. For each method 10 threads were launched; the figure shows at every instant the best value found by the threads.

To justify our choice of the eSS algorithm among the existing metaheuristics, we compare it with another state of the art metaheuristic such as Differential Evolution (DE). To ensure a fair comparison we set the same computation time as for the eSS and local multistart procedures: we carry out 10 DE optimizations, each with a time limit of one day. The convergence curves of the DE and eSS algorithms are shown in Figure 4, which plots, for each method, the best solution found by any of its 10 threads at every time instant (note that this is different from showing 10 different convergence curves for each method). Both algorithms obtain similar results in the first few hours; after that time, however, eSS achieves fast and significant improvements whereas DE progresses more slowly. This is due to the lack of local searches, which in this problem are instrumental in refining the solution. Hence, we see that for calibrating this model it is advisable to combine the intelligent exploration of the parameter space provided by metaheuristics with the refinement of the solution provided by local searches. This supports the choice of the eSS algorithm.

**Figure 5:**
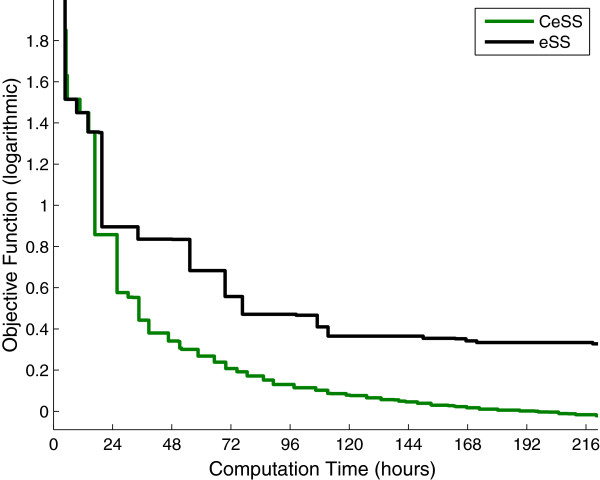
**Model 1 - Convergence curves, CeSS vs. eSS.** The performance of 10 individual, non cooperative threads (in black) is compared with *η *= 10 cooperative threads that exchange information with *τ *= 2.53 (in green). The figure shows the logarithm of the objective function value plotted against the computation time. For each method, only the best value found by its 10 threads is shown at every instant.

The next step is to improve the eSS results by means of the parallel cooperative strategy presented in the previous section, CeSS. With this aim, the number of threads is fixed as *η *= 10; each of them implement the eSS algorithm with different options (*dim_refset*, *local.n2*, and *balance*; the fourth option *ndiverse* is common to all threads). In this way each of the 10 threads has a different degree of aggressiveness. Figure 5 shows the convergence curves, which plot the logarithm of the objective function value against the computation time. The performance of the 10 individual, non cooperative threads (black line) is compared to the performance of the 10 cooperative threads (green line), which exchange information with *τ *= 2.53 (approximately every 12 hours in the hardware used). The results show that cooperation greatly speeds up the convergence: while the non-cooperative threads need 11 days to reach an objective function value of 1.88, the cooperative threads obtain the same result in approximately 1.5 days. Therefore cooperation reduces computation times by more than 85%. In 11 days, the cooperative threads achieve objective function values ranging between 8.05·10^−1^ and 9.71·10^−1^, thus improving the non-cooperative solution by more than 50%. More figures showing the influence of the tuning parameters *η*, *τ* are included as supplementary material, see Additional file 2.

**Figure 6:**
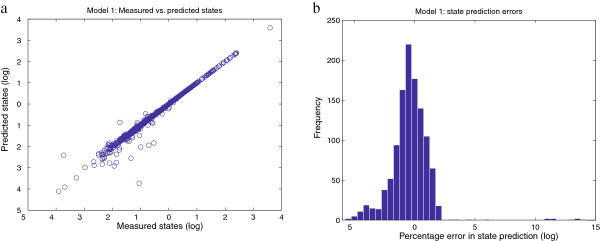
**Model 1 - relative errors in the model predictions. Figure**6**.a** plots the values of the measured vs. predicted states of the model. Note that the plot range is from 10^−5^to 10^−4^; 10 of the 1150 points have values less than 10^−6^and are not shown. **Figure**6**.b** shows an histogram of the residuals, which are calculated as $\left|(\tilde{x}-x)/x\right|\cdot 100$, where the predicted values are $\tilde{x}$. In both figures the results are plotted in a logarithmic scale.

**Figure 7:**
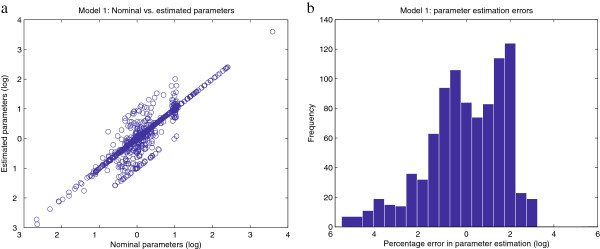
**Model 1 - relative errors in the estimated parameters.Figure**7**.a** plots the values of the nominal vs. estimated parameters. **Figure**7**.b** shows an histogram of the residuals, which are calculated as $\left|(\tilde{p}-p)/p\right|\cdot 100$, where the estimated parameters are $\tilde{p}$. In both figures the results are plotted in a logarithmic scale.

To give a better idea of the goodness of the fit, we test the ability of the calibrated model to reproduce the original data. Figure 6 plots in logarithmic scale the percentage error in the estimated values of the observables, which are concentrations and fluxes. Similarly, Figure 7 shows the errors in the estimated values of the parameters. It can be noticed that the model predictions for the observables (Figure 6.a) are better than the fit between the nominal and estimated parameters (Figure 7.a). That is, even though the optimization algorithm manages to fit the model predictions to the data quite nicely, there are some groups of parameters that are not estimated accurately. This fact reveals a lack of practical identifiability for the given model and data. The present work focuses on the performance of optimization algorithms; that is, in their ability to reduce the objective function value. This is different to the identifiability problem, which is not addressed here but can be surmounted by proper design of additional experiments. However, from the point of view of model calibration, the method presented here was able to successfully solve the inverse problem with better performance than other state of the art methods.

### Model 2: E. coli metabolic model including enzymatic and transcriptional regulation

The model calibrated in the previous subsection was purely metabolic. Here we consider a previously published *E. coli* model [18] that also takes into account the enzymatic and transcriptional regulation layer. It consists of 47 ODEs and 193 parameters (affinity constants, specific activities, Hill coefficients, growth rates, expression rates, etc), of which 178 are considered unknown and need to be estimated. We have reformulated the model to use it in an optimization context, where the objective function to be minimized consists of the difference between the simulated concentration profiles obtained with the nominal and the estimated parameters. Upper and lower bounds for the parameters are fixed to values 10 times larger and 10 times smaller than the nominal, except for the Hill coefficients, which are assumed to be between 0.5 and 4. More information is included as supplementary material, see Additional file 3.

This model was created to reproduce the way in which *E. coli* adapts to changing carbon sources. With this aim, it is subjected to a sequence of three consecutive environments, where the carbon source is first glucose, then acetate, and finally a mixture of both. Under these conditions, the 47 concentration profiles are sampled every 1000 seconds, for a total of 162 time points (45 hours). Therefore the overall number of available samples is 47 × 162 = 7614. The equations are integrated using LSODE (Livermore Solver for Ordinary Differential Equations) [36], which is suited for stiff systems. Integrating these equations is a computationally expensive task; one evaluation of the objective function takes several seconds.

It must be noted that in [18] a divide-and-conquer approach was adopted for estimating the parameter values of the rate equations. In this approach, the large-scale optimization problem is divided into a set of small subproblems, where only a few parameters are estimated at a time. This allows to reduce the complexity of the task and consequently the computation times. However, it is an ad hoc procedure that cannot be applied to most systems biology problems. Here we adopt a general purpose, large-scale global optimization approach that does not require any special structure of the model equations.

**Figure 8:**
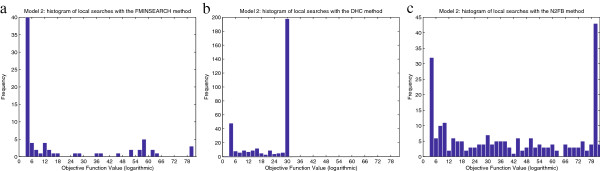
**Model 2 - Histograms of multistart local searches.** Objective function values achieved by local searches starting from different points with the FMINSEARCH **(Figure**8**.a)**, DHC **(Figure**8**.b)**, and N2FB **(Figure**8**.c)** methods. Calculations were carried out in a single processor; the computation time was 240 hours for each method. The best values obtained were: 3.046·10^3^(FMINSEARCH), 7.601·10^2^(DHC), and 1.3863·10^3^(N2FB). Note that the x axis is logarithmic in order to represent the large dispersion of the results.

As with Model 1, the calibration procedure begins by carrying out several multistart procedures of local methods. The selected methods are DHC, *fminsearch*, and N2FB (which outperforms *fmincon* for this problem). We limit the number of evaluations in the *fminsearch* method to 2000, so that each local search lasts typically around five hours on our computers; without this limit it can go on for much longer without achieving substantial improvements. The maximum time allowed for each multistart is ten days for all of the methods. Notice that, as with Model 1, each of the algorithms carries out a different number of local searches, despite the same computational time. The best solution is *f*_*best *_= 7.60·10^2^, found by the DHC method; an histogram of the results is shown in Figure 8.

Next, the enhanced Scatter Search (eSS) algorithm is tested. For this particular problem it was decided to use eSS with DHC as the local solver because, once again, it offers the best balance between quality of the solution and computational time. After selecting 10 different settings and letting the corresponding 10 optimizations run for 24 hours, the best objective function value achieved is *f*_*best *_= 2.37·10^2^ and the worst is *f*_*best *_= 1.48·10^3^. Thus the eSS algorithm clearly outperforms any local method.

**Figure 9:**
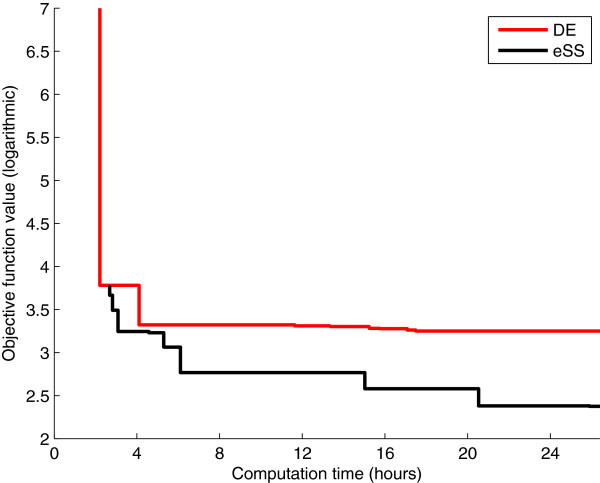
**Model 2 - Differential Evolution vs. non-cooperative enhanced Scatter Search.** Convergence curves of the DE and eSS methods. For each method 10 threads were launched; the figure shows at every instant the best value found by the threads.

Again, we compare the performance of the eSS algorithm with the differential evolution metaheuristic (DE). We carry out 10 DE optimizations, each with a time limit of 24 hours, and do the same comparison of convergence curves performed with Model 1. As can be seen in Figure 9, eSS also outperforms DE for this particular problem.

**Figure 10:**
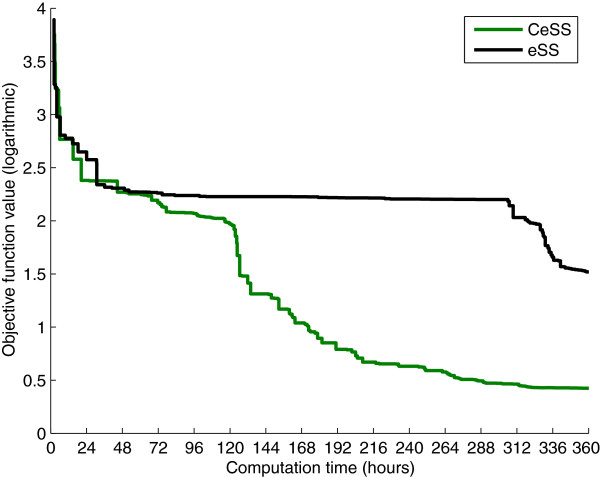
**Model 2 - Convergence curves, CeSS vs. eSS.** The logarithm of the objective function value is plotted against the computation time. The performance of 10 individual, non cooperative threads is shown in black. It is compared with *η *= 10 cooperative threads that exchange information with *τ *= 2.15 (green line). For each method, only the best value found by its 10 threads is shown at every instant

**Figure 11:**
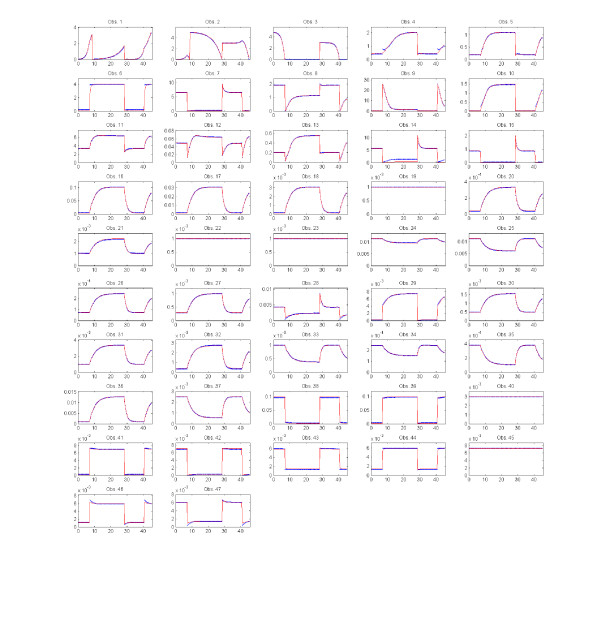
**Model 2 - Data vs. model predictions.** Dynamics of the 47 model states. Data points used for calibration are shown as blue dots; the calibrated model predictions are shown as red lines. The plots show a near perfect match between data and predictions.

**Figure 12:**
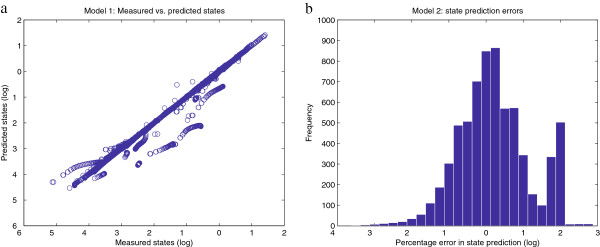
**Model 2 - relative errors in the model predictions.Figure**12**.a** plots the values of the measured vs. predicted states of the model. For clarity, values corresponding to very small concentrations (<10^−6^) are not shown. **Figure**12**.b** shows an histogram of the residuals, which are calculated as $\left|(\tilde{x}-x)/x\right|\cdot 100$, where the predicted values are $\tilde{x}$.

**Figure 13:**
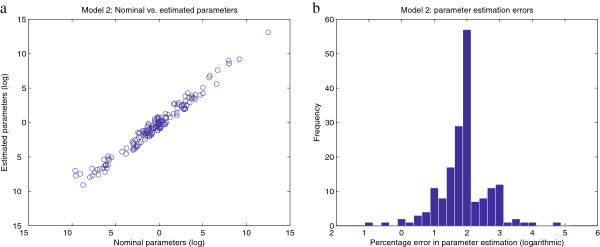
**Model 2 - relative errors in the estimated parameters. Figure**13**.a** plots the values of the nominal vs. estimated parameters. **Figure**13**.b** shows an histogram of the residuals, which are calculated as $\left|(\tilde{p}-p)/p\right|\cdot 100$, where the estimated parameters are $\tilde{p}$. In both figures the results are plotted in a logarithmic scale. The 11 parameters with values <10^−50^are not shown.

Then we test the cooperative optimization method, CeSS. With this aim, we launch *η*=10 cooperative threads, each of them implementing the eSS algorithm with different options (*dim_refset*, *local.n2*, *balance*); the interval between information sharing is *τ *= 2.15, which in the hardware used corresponds to 24 hours. Figure 10 plots the convergence curves showing the best value found at every instant by the 10 cooperative threads compared to that found by 10 non-cooperative threads. As with Model 1, it can be seen that cooperation speeds up convergence. After 17 days CeSS reaches an objective function value of 2.51, while eSS obtains 2.58·10^1^. The solution retrieved by CeSS represents a very good match between data and model predictions, as shown in Figure 11. Figure 12 plots in logarithmic scale the percentage error in the estimated values of the observables, while Figure 13 shows the errors in the estimated values of the parameters; these plots reveal practical identifiability issues similar to those detected in Model 1. In this case, identifiability issues were actually expected, given that (i) some parameter values in the originally published model [18] are very different from those in the version available in the BioModels database [37] (model ID: 244), but (ii) despite the differences, both parameter sets yield similar simulation results. This is a clear sign of lack of identifiability. However, once again, the new method is capable of successfully solving the calibration problem, which is the main objective of this work. The practical identifiability problems are a consequence of lack of information in the considered data set and can be surmounted by proper design of additional experiments [4].

Finally, we would like to comment on the default values recommended for the search parameters of the cooperative strategy. The main potential disadvantage of using such default settings for a new problem is that the method could perform worse in two extreme situations: easy (mildly non-convex) problems, where a simple multi-start local method could perform very well, and extremely hard (highly non-convex problems) where there is no structure at all and local searches do not help. Since for each new problem we do not have any a priori information about the topology of the search space, we simply recommend a broad spectrum of values for the aggressiveness of the threads, so even in the extreme situations the cooperative strategy would be able to solve the problem. Of course, any a posteriori information about the topology, after a few runs with the default settings, can be exploited by tuning *ψ*. This reasoning applies for the case of new arbitrary problems, but we should add that for the particular large-scale parameter estimation problems considered above, the default recommended values have shown a good performance.

## Conclusions

### Summary

Typically, systems biology models have a large number of parameters and are highly nonlinear. Furthermore, the information content of the available experimental data is frequently scarce. As a consequence, it is hard to find the set of parameter values that gives the best fit to experimental data. This task, known as model calibration, is commonly formulated as an optimization problem, which in these cases must be solved with global optimization techniques.

Here we have presented a new method for solving this problem, called Cooperative enhanced Scatter Search (CeSS), which is specifically designed to profit from a parallel environment with several processors. Each of the processors executes a program (or “thread”) which implements the enhanced Scatter Search (eSS) algorithm [24,25]. This is a state of the art metaheuristic capable of competing succesfully with other global optimization methods. The key feature of the strategy presented here is the cooperation between threads, which means that they exchange information at certain fixed instants. This information consists of the reference set of solutions they have found so far; it depends on the best solution found, the shape of the solution space, and the settings of each algorithm.

We have tested this strategy with two large-scale *E. coli* models of different sizes and characteristics. The first one models the central carbon metabolism (CCM) using a recently proposed common modular rate law [11]. The second is a previously published model that combines metabolism with a transcriptional regulatory layer [18]. In both cases the presented method clearly outperformed several state of the art techniques. The performance and capabilities of the method have also been evaluated using benchmark problems of large-scale global optimization, with excellent results.

### Insights

The results for the cooperative strategy presented here shows how cooperation of individual parallel search threads modifies the systemic properties of the individual algorithms, improving its performance and outperforming other competitive methods. Importantly, it is a general purpose framework that can be easily applied to any model calibration problem. Furthermore, the underlying cooperative parallel mechanism is relatively simple and can be extended to incorporate other global and local search solvers and specific structural information for particular classes of problems.

## Competing interests

Herewith we, the authors, confirm that we have no competing interests.

## Authors’ contributions

All authors contributed to the conception and design of the work. AFV performed the numerical computations. All authors contributed to the writing of the manuscript. All authors read and approved the final manuscript.

## Supplementary Material

Additional file 1 **LSGO Benchmark.** The file includes tests of the eSS and CeSS methods with the Large-Scale Global Optimization benchmark ( http://staff.ustc.edu.cn/∼ketang/cec2012/lib/lsgo_benchmark.zip) [38,39]. Click here for file

Additional file 2 **Model 1.** The file includes tables listing the model reactions and metabolites (KEGG IDs are given when available), nominal values of the parameters, experimental conditions, and additional convergence curves showing the algorithm’s performance. Model supplied by Wolfram Liebermeister (personal communication). Click here for file

Additional file 3 **Model 2.** The file includes information about the model structure and parameters, plots of the fits between the calibrated model and the simulated experimental data, and additional convergence curves showing the algorithm’s performance [ [18]]. Click here for file
