# Supplementary material for: A cooperative strategy for parameter estimation in large scale systems biology models
Source: BMC Syst Biol. 2012 Jun 22;6:75. doi: 10.1186/1752-0509-6-75 (PMC3512509; doi:10.1186/1752-0509-6-75)
Supplement: Additional file 1 — LSGO Benchmark. The file includes tests of the eSS and CeSS methods with the Large-Scale Global Optimization benchmark ( http://staff.ustc.edu.cn/∼ketang/cec2012/lib/lsgo_benchmark.zip) [38,39]. [file 1752-0509-6-75-S1.pdf]

Large-Scale Global Optimization Benchmark

In this document CeSS is applied to a test function taken from a well-known benchmark for large-scale global optimization algorithms. This test serves as a proof of concept that the method is suitable for large-scale problems, which need not be necessarily biological.

1 The LSGO benchmark

The competition on Large Scale Global Optimization (LSGO) is organized in company with the Special Session on Evolutionary Computation for Large Scale Global Optimization, which will be part of the 2012 IEEE World Congress on Computational Intelligence (CEC@WCCI-2012) to be held in June 10-15, 2012, Brisbane, Australia. This is the most recent of a series of challenges that have also been organized in the 2008 and 2010 conferences. The set of benchmark functions used for the 2012 edition, which is the same that was used in the previous (2010) conference, can be downloaded from `http://staff.ustc.edu.cn/~ketang/cec2012/lib/lsgo_benchmark.zip`. A total number of 20 functions of different characteristics are included in the set. There are 5 categories:

- Separable functions (F1–F3)
- Single-group m-nonseparable functions (F4–F8)
- D/2m-group m-nonseparable functions (F9–F13)
- D/m-group m-nonseparable functions (F14-F18)
- Nonseparable functions (F19–F20)

where D is the dimension, $D = 1000$, and m is the grouping structure ($m = 50$, when applicable).

2 Test of eSS with the LSGO benchmark

Finding the global optimum of the aforementioned functions is a notoriously difficult task. To give an idea of the performance that can be expected from a state of the art method when applied to this problem, the competition webpage (`http://staff.ustc.edu.cn/~ketang/cec2012/lsgo_competition.htm`) gives results obtained with three methods published in 2008: DECC-G [1], DECC-G* (a modification of DECC-G), and MLCC [2]. Each method is run 25 times, and a maximum of $3 \cdot 10^6$ function evaluations is allowed. It must be noted that the DECC-G* method uses the grouping structure ($m = 50$) as previous

knowledge. Since the use of this information for design purposes is not allowed in the LSGO competition, DECC-G* should be regarded as un “unfair” competitor. Table 1 compare the results reported with these methods with those obtained by the non-cooperative version of CeSS, eSS, using functions F10–F20 as case studies. Table 2 ranks the optimization methods according to their performances; the following conclusions can be extracted:

1. No algorithm performs best with all of the functions.
2. On average, DECC-G* is the best performer; however, it uses grouping information, which is forbidden for the benchmark participants.
3. On average, eSS outperforms the other two methods, DECC-G and MLCC.

We conclude that eSS performs well compared with other state-of-the-art methods, and is therefore a suitable technique for solving LSGO problems.

Table 1: Optimization results for F10–F20 with several methods

**F10**	DECC-G	DECC-G*	MLCC	eSS
Best	$1.03 \cdot 10^4$	$2.33 \cdot 10^3$	$2.52 \cdot 10^3$	$5.57 \cdot 10^3$
Median	$1.07 \cdot 10^4$	$2.49 \cdot 10^3$	$3.16 \cdot 10^3$	$5.96 \cdot 10^3$
Worst	$1.17 \cdot 10^4$	$2.64 \cdot 10^3$	$5.90 \cdot 10^3$	$6.26 \cdot 10^3$
Mean	$1.06 \cdot 10^4$	$2.48 \cdot 10^3$	$3.43 \cdot 10^3$	$5.94 \cdot 10^3$
Std	$2.95 \cdot 10^2$	$7.63 \cdot 10^1$	$8.72 \cdot 10^2$	$1.88 \cdot 10^2$
**F11**	DECC-G	DECC-G*	MLCC	eSS
Best	$2.06 \cdot 10^1$	$5.82 \cdot 10^{-8}$	$1.96 \cdot 10^2$	$1.93 \cdot 10^2$
Median	$2.33 \cdot 10^1$	$7.52 \cdot 10^{-8}$	$1.98 \cdot 10^2$	$1.95 \cdot 10^2$
Worst	$2.79 \cdot 10^1$	$8.79 \cdot 10^{-1}$	$1.98 \cdot 10^2$	$1.96 \cdot 10^2$
Mean	$2.34 \cdot 10^1$	$3.52 \cdot 10^{-2}$	$1.98 \cdot 10^2$	$1.95 \cdot 10^2$
Std	$1.78 \cdot 10^0$	$1.76 \cdot 10^{-1}$	$6.98 \cdot 10^{-1}$	$6.09 \cdot 10^{-1}$
**F12**	DECC-G	DECC-G*	MLCC	eSS
Best	$7.78 \cdot 10^4$	$6.16 \cdot 10^1$	$2.42 \cdot 10^4$	$1.97 \cdot 10^4$
Median	$8.87 \cdot 10^4$	$7.72 \cdot 10^1$	$3.47 \cdot 10^4$	$2.93 \cdot 10^4$
Worst	$1.07 \cdot 10^5$	$1.19 \cdot 10^2$	$4.25 \cdot 10^4$	$4.79 \cdot 10^4$
Mean	$8.93 \cdot 10^4$	$7.87 \cdot 10^1$	$3.49 \cdot 10^4$	$3.15 \cdot 10^4$
Std	$6.87 \cdot 10^3$	$1.41 \cdot 10^1$	$4.92 \cdot 10^3$	$8.46 \cdot 10^3$
**F13**	DECC-G	DECC-G*	MLCC	eSS
Best	$1.78 \cdot 10^3$	$3.78 \cdot 10^2$	$1.01 \cdot 10^3$	$6.12 \cdot 10^2$
Median	$3.00 \cdot 10^3$	$5.40 \cdot 10^2$	$1.91 \cdot 10^3$	$1.02 \cdot 10^3$
Worst	$1.66 \cdot 10^4$	$7.55 \cdot 10^2$	$3.47 \cdot 10^3$	$2.51 \cdot 10^3$
Mean	$5.12 \cdot 10^3$	$5.50 \cdot 10^2$	$2.08 \cdot 10^3$	$1.17 \cdot 10^3$
Std	$3.95 \cdot 10^3$	$9.78 \cdot 10^1$	$7.27 \cdot 10^2$	$4.81 \cdot 10^2$
**F14**	DECC-G	DECC-G*	MLCC	eSS
Best	$6.96 \cdot 10^8$	$2.46 \cdot 10^7$	$2.62 \cdot 10^8$	$2.37 \cdot 10^7$
Median	$8.07 \cdot 10^8$	$2.90 \cdot 10^7$	$3.16 \cdot 10^8$	$3.31 \cdot 10^7$
Worst	$9.06 \cdot 10^8$	$3.56 \cdot 10^7$	$3.77 \cdot 10^8$	$4.68 \cdot 10^7$

Mean	$8.08 \cdot 10^8$	$2.91 \cdot 10^7$	$3.16 \cdot 10^8$	$3.29 \cdot 10^7$
Std	$6.07 \cdot 10^7$	$2.91 \cdot 10^6$	$2.77 \cdot 10^7$	$6.15 \cdot 10^6$
**F15**	DECC-G	DECC-G*	MLCC	eSS
Best	$1.09 \cdot 10^4$	$3.62 \cdot 10^3$	$5.30 \cdot 10^3$	$6.84 \cdot 10^3$
Median	$1.18 \cdot 10^4$	$3.88 \cdot 10^3$	$6.89 \cdot 10^3$	$7.71 \cdot 10^3$
Worst	$1.39 \cdot 10^4$	$4.25 \cdot 10^3$	$1.04 \cdot 10^4$	$8.09 \cdot 10^3$
Mean	$1.22 \cdot 10^4$	$3.88 \cdot 10^3$	$7.11 \cdot 10^3$	$7.71 \cdot 10^3$
Std	$8.97 \cdot 10^2$	$1.76 \cdot 10^2$	$1.34 \cdot 10^2$	$2.36 \cdot 10^2$
**F16**	DECC-G	DECC-G*	MLCC	eSS
Best	$5.97 \cdot 10^1$	$7.04 \cdot 10^{-8}$	$2.08 \cdot 10^2$	$3.83 \cdot 10^2$
Median	$7.51 \cdot 10^1$	$1.04 \cdot 10^{-7}$	$3.95 \cdot 10^2$	$3.84 \cdot 10^2$
Worst	$9.24 \cdot 10^1$	$2.18 \cdot 10^0$	$3.97 \cdot 10^2$	$3.85 \cdot 10^2$
Mean	$7.66 \cdot 10^1$	$4.01 \cdot 10^{-1}$	$3.76 \cdot 10^2$	$3.84 \cdot 10^2$
Std	$8.14 \cdot 10^0$	$6.59 \cdot 10^{-1}$	$4.71 \cdot 10^1$	$5.58 \cdot 10^{-1}$
**F17**	DECC	DECC-G*	MLCC	eSS
Best	$2.50 \cdot 10^5$	$8.09 \cdot 10^1$	$1.38 \cdot 10^5$	$4.48 \cdot 10^4$
Median	$2.89 \cdot 10^5$	$1.03 \cdot 10^2$	$1.59 \cdot 10^5$	$7.18 \cdot 10^4$
Worst	$3.26 \cdot 10^5$	$1.33 \cdot 10^2$	$1.86 \cdot 10^5$	$9.65 \cdot 10^4$
Mean	$2.87 \cdot 10^5$	$1.03 \cdot 10^2$	$1.59 \cdot 10^5$	$7.16 \cdot 10^4$
Std	$1.98 \cdot 10^4$	$1.38 \cdot 10^1$	$1.43 \cdot 10^4$	$1.59 \cdot 10^4$
**F18**	DECC-G	DECC-G*	MLCC	eSS
Best	$5.61 \cdot 10^3$	$8.37 \cdot 10^2$	$2.51 \cdot 10^3$	$1.46 \cdot 10^3$
Median	$2.30 \cdot 10^4$	$1.08 \cdot 10^3$	$4.17 \cdot 10^3$	$2.39 \cdot 10^3$
Worst	$4.71 \cdot 10^4$	$1.53 \cdot 10^3$	$1.62 \cdot 10^4$	$5.61 \cdot 10^3$
Mean	$2.46 \cdot 10^4$	$1.08 \cdot 10^3$	$7.09 \cdot 10^3$	$2.73 \cdot 10^3$
Std	$1.05 \cdot 10^4$	$1.61 \cdot 10^2$	$4.77 \cdot 10^3$	$1.04 \cdot 10^3$
**F19**	DECC-G	DECC-G*	MLCC	eSS
Best	$1.02 \cdot 10^6$	$9.90 \cdot 10^5$	$1.21 \cdot 10^6$	$6.78 \cdot 10^5$
Median	$1.11 \cdot 10^6$	$1.15 \cdot 10^6$	$1.36 \cdot 10^6$	$1.09 \cdot 10^6$
Worst	$1.20 \cdot 10^6$	$1.23 \cdot 10^6$	$1.54 \cdot 10^6$	$1.58 \cdot 10^6$
Mean	$1.11 \cdot 10^6$	$1.14 \cdot 10^6$	$1.36 \cdot 10^6$	$1.04 \cdot 10^6$
Std	$5.15 \cdot 10^4$	$5.85 \cdot 10^4$	$7.35 \cdot 10^4$	$2.74 \cdot 10^5$
**F20**	DECC	DECC-G*	MLCC	eSS
Best	$3.59 \cdot 10^3$	$2.83 \cdot 10^3$	$1.70 \cdot 10^3$	$9.74 \cdot 10^2$
Median	$3.98 \cdot 10^3$	$3.21 \cdot 10^3$	$2.04 \cdot 10^3$	$9.85 \cdot 10^2$
Worst	$5.32 \cdot 10^3$	$6.23 \cdot 10^3$	$2.34 \cdot 10^3$	$1.04 \cdot 10^3$
Mean	$4.06 \cdot 10^3$	$3.33 \cdot 10^3$	$2.05 \cdot 10^3$	$9.97 \cdot 10^2$
Std	$3.66 \cdot 10^2$	$6.63 \cdot 10^2$	$1.80 \cdot 10^2$	$2.28 \cdot 10^1$

Table 2: Algorithms performance

Method	F_{10}	F_{11}	F_{12}	F_{13}	F_{14}	F_{15}	F_{16}	F_{17}	F_{18}	F_{19}	F_{20}
DECC-G*	1	1	1	1	1	1	1	1	1	2	3
eSS	3	3	2	2	2	3	3	2	2	1	1
MLCC	2	4	3	3	3	2	3	3	3	4	2
DECC-G	4	2	4	4	4	4	2	4	4	3	4

3 Test of CeSS with the LSGO benchmark

In this section we evaluate the performance of CeSS, in order to assess the influence of cooperation. We have selected as case studies three of the benchmark functions, each one belonging to a different category. We launch for each function 10 cooperative threads and allow for 20 cooperation instants, one every hour. We compare the results with 10 non-cooperative threads, that is, threads that implement the eSS algorithm alone. To account for the variability due to the stochastic nature of the algorithm, we repeat the computations 10 times. Thus the overall number of threads (in the cooperative as well as in the non-cooperative case) is 100. For a meaningful comparison we divide the 100 individual, non-cooperative threads in 10 groups of 10 threads; each group can thus be compared to a set of 10 cooperative threads. The best objective function value found at every moment by any of the 10 non-cooperative threads in each group should be compared with the best objective function value found at every moment by any of the 10 threads in a cooperative group. Hence we have 10 cooperative and 10 non-cooperative convergence curves. The following subsections describe the selected functions and show the optimization results.

3.1 F_{10}: D/2m-group Shifted and m-rotated Rastrigin's function

The original Rastrigin's function is

$$F_{rastrigin}(\mathbf{x}) = \sum_{i=1}^{D} \left[x_i^2 - 10cos(2\pi x_i) + 10\right] \quad (1)$$

where the dimension is $D = 1000$ and $\mathbf{x} = (x_1, x_2, \ldots, x_D)$ is the candidate solution (a D-dimensional vector). This is a separable function; to make it unseparable, a coordinate rotation is performed, yielding the rotated Rastrigin's function:

$$F_{rot-rastrigin}(\mathbf{x}) = F_{rastrigin}(\mathbf{z}),\ \mathbf{z} = \mathbf{x} * \mathbf{M} \quad (2)$$

where M is a $D \times D$ orthogonal matrix. Rastrigin's function is a classical multimodal problem; it is difficult since the number of local optima grows exponentially with the increase of dimensionality. Finally, F_{10} is composed of the original and the rotated Rastrigin's functions:

$$F_{10}(\mathbf{x}) = \sum_{k=1}^{\frac{D}{2m}} F_{rot-rastrigin}\left[z\left(P_{(k-1)*m+1} : P_{k*m}\right) + F_{rastrigin}\left[z\left(P_{\frac{D}{2}+1} : P_D\right)\right]\right. \quad (3)$$

where $m = 50$ is the group size; $\mathbf{o} = (o_1, o_2, \ldots, o_D)$ is the shifted global optimum; $\mathbf{z} = \mathbf{x}$ - $\mathbf{o}$ is the shifted candidate solution; and P is a random permutation of 1,2,...,D. This function has the following properties:

1. Multimodal
2. Shifted
3. D/2m-group m-rotated
4. D/2m-group m-nonseparable
5. $x \in [-5, 5]^D$
6. Global optimum: x* = **o**, $F_{10}(\mathbf{x}*) = 0$

3.2 F_{17}, D/m-group Shifted m-dimensional Schwefel's problem 1.2

Schwefel's Problem 1.2 is a naturally nonseparable function, which is defined as follows:

$$F_{schwefel}(\mathbf{x}) = \sum_{i=1}^{n} \left(\sum_{j=1}^{i} x_i \right)^2 \tag{4}$$

where D is the dimension and x=$(x_1, x_2, \ldots, x_D)$ is a D-dimensional row vector. The benchmark function F_{17} is the D/m-group Shifted m-dimensional version of $F_{schwefel}$, that is,

$$F_{17}(x) = \sum_{k=1}^{\frac{D}{m}} F_{schwefel} \left[\mathbf{z} \left(P_{(k-1)*m+1} : P_{k*m} \right) \right] \tag{5}$$

where the notation is the same as in F_{10}. This function has the following properties:

1. Unimodal
2. Shifted
3. D/m-group m-nonseparable
4. $x \in [-100, 100]^D$
5. Global optimum: x* = **o**, $F_{17}(\mathbf{x}*) = 0$

3.3 F_{20}, the shifted Rosenbrocks function

$$F_{20}(\mathbf{x}) = \sum_{i=1}^{D-1} \left[100 \left(z_i^2 - z_{i+1} \right)^2 + (z_i - 1)^2 \right] \tag{6}$$

where the dimension is $D = 1000$; $\mathbf{x} = (x_1, x_2, \ldots, x_D)$ is the candidate solution (a D-dimensional vector); $\mathbf{o} = (o_1, o_2, \ldots, o_D)$ is the shifted global optimum; and $\mathbf{z} = \mathbf{x}$ - $\mathbf{o}$ is the shifted candidate solution. This function has the following properties:

1. Multimodal.
2. Shifted.
3. Fully non-separable, that is, any two of its parameters are not independent.
4. Parameter bounds: [-100,100].
5. Global optimum: $\mathbf{x}* = \mathbf{o} + 1$, $F_{20}(\mathbf{x}*) = 0$.

3.4 Results for F10, F17, and F20, eSS vs. CeSS

Figures 1–3 compare the optimization results obtained for functions F10, F17, and F20, respectively, by eSS and CeSS. The performance of 100 individual (non-cooperative) threads is compared to that of 100 threads that cooperate in groups of 10. For a meaningful comparison, the 100 individual threads are grouped in subsets of 10 threads. The best objective function value found at every moment by any of the 10 non-cooperative threads is compared with the best objective function value found at every moment by any of the 10 cooperative threads.

It can be seen that for the three functions the cooperative version, CeSS (black lines) outperforms on average the non-cooperative one, eSS (blue lines). The clearest advantage happens with F20. However, it should be noted that not all of the cooperative groups outperform all of the non-cooperative groups at all times. This remark reminds us of the stochastic nature of the algorithm. This conclusion is common for the three functions.

4 Influence of the τ parameter in CeSS

In the previous sections we have shown that (i) the core algorithm, enhanced scatter search (eSS) performs well with functions from a Large-Scale Global Optimization benchmark; and that (ii) this performance is clearly improved when a cooperative strategy is implemented, transforming eSS into cooperative eSS (CeSS). In this section we test the influence of the interval between cooperation instants, τ, in the algorithm's performance. For this purpose we use function F20. Figures 3–5 show optimization results for $\tau = 3.9$, 4.2 and 4.5, respectively, which corresponds to a time interval of 1, 2 and 4 hours in the hardware

used. Since the algorithm is allowed to perform a sufficient number of iterations between cooperation instants in all three cases, it works well in all of them and the differences in performance are small.

References

1. Yang Z, Tang K, Yao X: **Large scale evolutionary optimization using cooperative coevolution**. *Information Sciences* 2008, **178**(15):2985–2999.

2. Yang Z, Tang K, Yao X: **Multilevel cooperative coevolution for large scale optimization**. In *Evolutionary Computation, 2008. CEC 2008.(IEEE World Congress on Computational Intelligence). IEEE Congress on*, IEEE 2008:1663–1670.

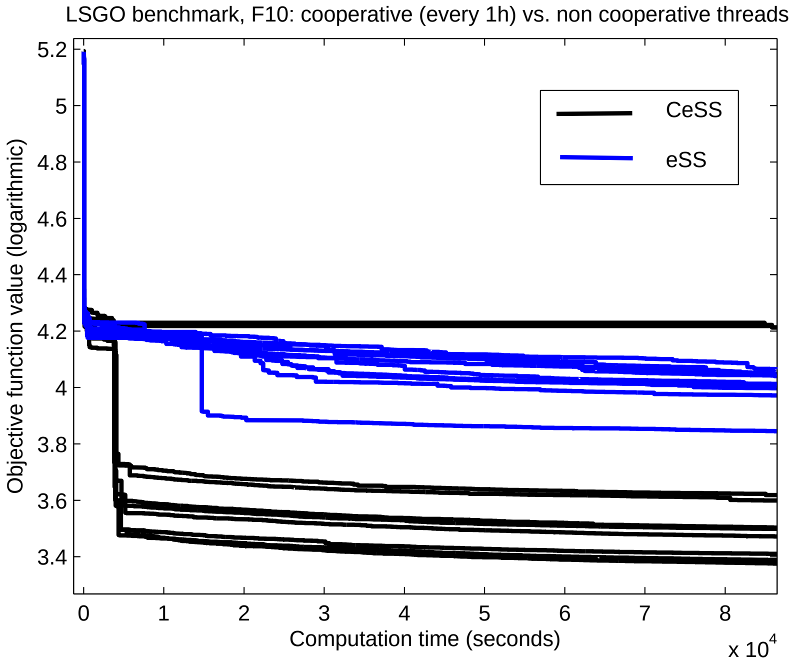

Figure 1: CeSS vs. eSS, F_{10}, $\tau = 3.5$ (approx. 1 hour with the hardware used).

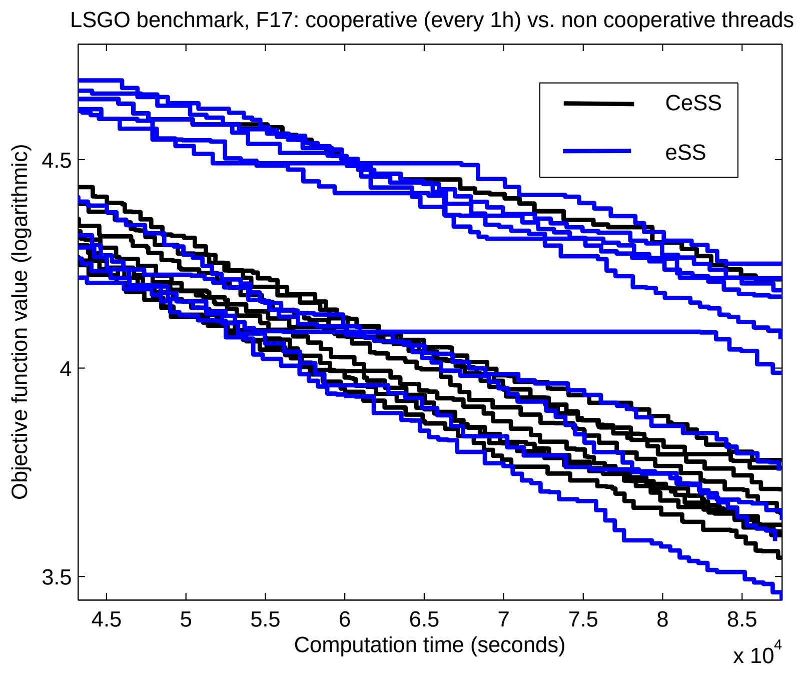

Figure 2: CeSS vs. eSS, F_{17}, $\tau = 2.8$ (approx. 1 hour with the hardware used).

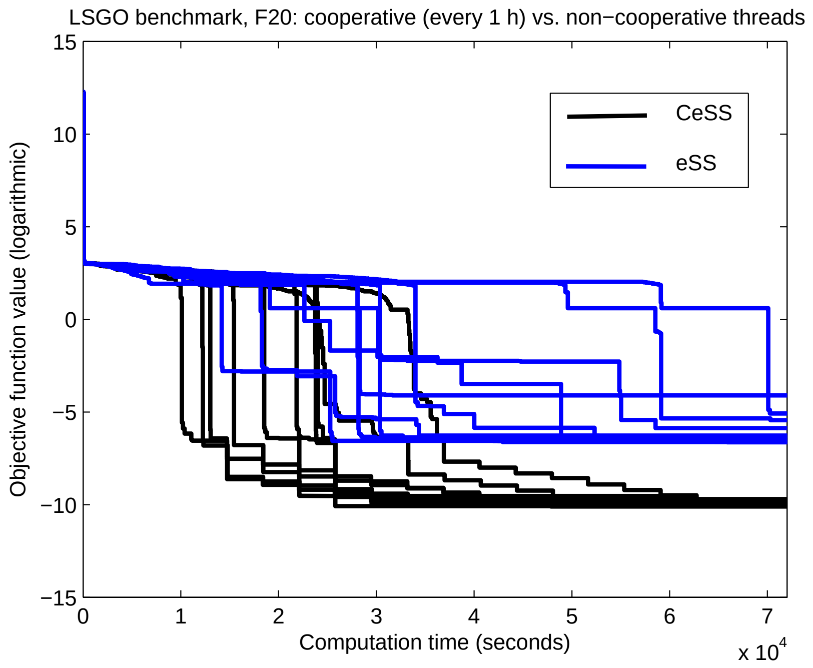

Figure 3: CeSS vs. eSS, F_{20}, $\tau = 3.9$ (approx. 1 hour with the hardware used).

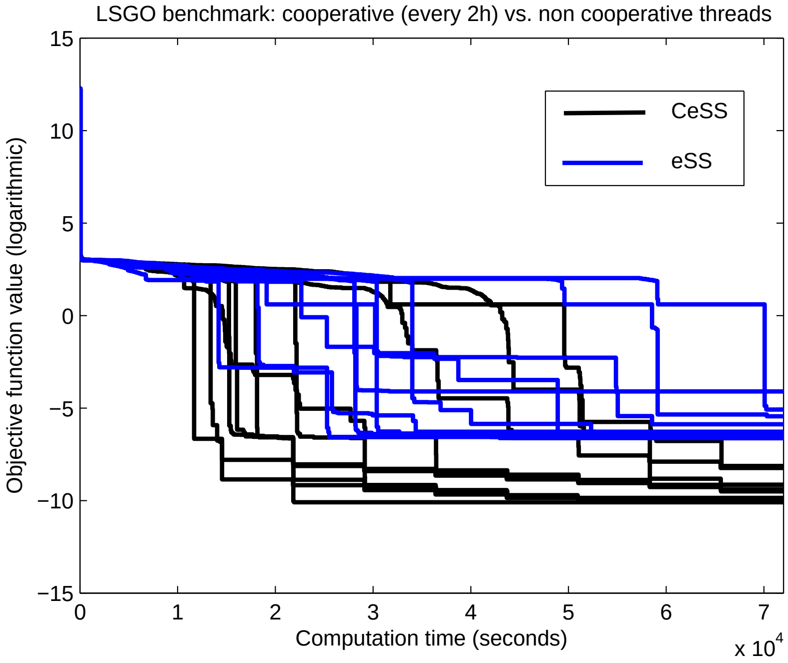

Figure 4: CeSS vs. eSS, F_{20}, $\tau = 4.2$ (approx. 2 hours with the hardware used)

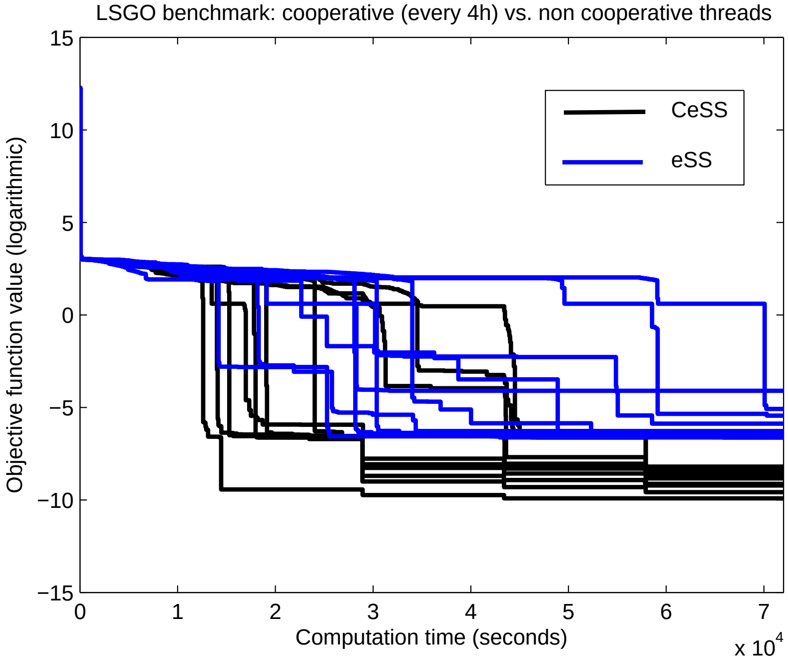

Figure 5: CeSS vs. eSS, F_{20}, $\tau = 4.5$ (approx. 4 hours with the hardware used)
