# Supplementary material for: A cooperative strategy for parameter estimation in large scale systems biology models
Source: BMC Syst Biol. 2012 Jun 22;6:75. doi: 10.1186/1752-0509-6-75 (PMC3512509; doi:10.1186/1752-0509-6-75)
Supplement: Additional file 2 — Model 1. The file includes tables listing the model reactions and metabolites (KEGG IDs are given when available), nominal values of the parameters, experimental conditions, and additional convergence curves showing the algorithm’s performance. Model supplied by Wolfram Liebermeister (personal communication). [file 1752-0509-6-75-S2.pdf]

MODEL 1

Sections 1 to 4 describe Model 1, a metabolic model of *E. coli*'s Central Carbon Metabolism. Section 5 provides additional information about the performance of the CeSS algorithm.

1 Model structure: metabolites and reactions

1.1 Metabolite list

Last column: 1 for external metabolites, 0 for internal.

#	Metabolite Name	KEGG ID	Ext. / Int.
1	Pyruvate	C00022	0
2	Thiamin diphosphat	C00068	1
3	2-(alpha-Hydroxyethyl)thiamine diphosphat	C05125	0
4	CO2	C00011	1
5	ATP	C00002	0
6	H2O	C00001	1
7	AMP	C00020	0
8	Phosphoenolpyruvat	C00074	0
9	Orthophosphat	C00009	1
10	ADP	C00008	0
11	Acetyl-CoA	C00024	1
12	Formate	C00058	1
13	CoA	C00010	1
14	(S)-Malate	C00149	0
15	NAD+	C00003	0
16	NADH	C00004	0
17	H+	C00080	1
18	NADP+	C00006	0
19	NADPH	C00005	0
20	Acetaldehyd	C00084	0
21	Acetyl phosphat	C00227	0
22	Acetate	C00033	1
23	Diphosphat	C00013	1
24	L-Glutamat	C00025	1
25	2-Oxoglutarat	C00026	0
26	NH3	C00014	1
27	Oxalosuccinat	C05379	0
28	Oxaloacetat	C00036	0
29	Citrate	C00158	0
30	Succinate	C00042	0
31	Succinyl-Co	C00091	0
32	Acceptor	C16722	0
33	Fumarate	C00122	0
34	Reduced accepto	C00030	0
35	Glyoxylate	C00048	0
36	Isocitrate	C00311	0
37	3-Carboxy-1-hydroxypropyl-ThP	C05381	0
38	2-Phospho-D-glycerat	C00631	0

39	(S)-Lactat	C00186	1
40	Ethanol	C00469	1
41	D-Glyceraldehyde 3-phosphat	C00118	0
42	Glycerone phosphat	C00111	0
43	D-Ribose 5-phosphat	C00117	0
44	5-Phospho-alpha-D-ribose 1-diphosphat	C00119	1
45	D-Ribulose 5-phosphat	C00199	0
46	3-Phospho-D-glyceroyl phosphat	C00236	0
47	beta-D-Fructose 1,6-bisphosphat	C05378	0
48	cis-Aconitat	C00417	0
49	3-Phospho-D-glycerat	C00197	0
50	6-Phospho-D-gluconat	C00345	0
51	D-Xylulose 5-phosphat	C00231	0
52	beta-D-Glucose	C00221	0
53	beta-D-Glucose 6-phosphat	C01172	0
54	Sedoheptulose 7-phosphat	C05382	0
55	Dihydrolipoamid	C00579	1
56	Lipoamide	C00248	1
57	D-Erythrose 4-phosphat	C00279	0
58	beta-D-Fructose 6-phosphat	C05345	0
59	D-Glucono-1,5-lactone 6-phosphat	C01236	0
60	2-Dehydro-3-deoxy-6-phospho-D-gluconat	C04442	0
61	Enzyme N6-(dihydrolipoyl)lysin	C15973	1
62	(Dihydrolipoyllysine-residue acetyltransferase)-S-acetyldihydrolipoyllysin	C16255	0
63	(Dihydrolipoyllysine-residue succinyltransferase)-S-succinyldihydrolipoyllysin	C16254	0
64	Ferricytochrome b	C00995	1
65	Ferrocytochrome b	C00998	1
66	Enzyme N6-(lipoyl)lysin	C15972	1
67	sn-Glycerol 3-phosphat	C00093	0
68	Glycerol	C00116	0
69	GlycerolEx	–	1
70	beta-D-GlucoseEx	–	1
71	GTP	C00044	0
72	GDP	C00035	0
73	O2	C00007	1
74	Biomass	–	1

1.2 Reaction list

#	Formula	KEGG ID
1	Pyruvate + Thiamin diphosphate ↔ 2-(alpha-Hydroxyethyl)thiamine diphosphate + CO2	R00014
2	Pyruvate + ATP + H2O ↔ AMP + Phosphoenolpyruvate + Orthophosphate	R00199
3	Pyruvate + ATP ↔ Phosphoenolpyruvate + ADP	R00200
4	Acetyl-CoA + Formate ↔ Pyruvate + CoA	R00212
5	(S)-Malate + NAD+ ↔ Pyruvate + CO2 + NADH + H+	R00214
6	(S)-Malate + NADP+ ↔ Pyruvate + CO2 + H+ + NADPH	R00216
7	CoA + NAD+ + Acetaldehyde ↔ Acetyl-CoA + NADH + H+	R00228
8	Orthophosphate + Acetyl-CoA ↔ CoA + Acetyl phosphate	R00230

9	ATP + CoA + Acetate ↔ AMP + Acetyl-CoA + Diphosphate	R00235
10	H2O + NAD+ + L-Glutamate ↔ NADH + H+ + 2-Oxoglutarate + NH3	R00243
11	Oxalosuccinate ↔ CO2 + 2-Oxoglutarate	R00268
12	ATP + Acetate ↔ ADP + Acetyl phosphate	R00315
13	ATP + Oxaloacetate ↔ CO2 + Phosphoenolpyruvate + ADP	R00341
14	(S)-Malate + NAD+ ↔ NADH + H+ + Oxaloacetate	R00342
15	CoA + Citrate ↔ H2O + Acetyl-CoA + Oxaloacetate	R00351
16	Citrate ↔ Acetate + Oxaloacetate	R00362
17	ATP + CoA + Succinate ↔ Orthophosphate + ADP + Succinyl-CoA	R00405
18	Succinate + Acceptor ↔ Fumarate + Reduced acceptor	R00412
19	CoA + (S)-Malate ↔ H2O + Acetyl-CoA + Glyoxylate	R00472
20	Isocitrate ↔ Succinate + Glyoxylate	R00479
21	Thiamin diphosphate + 2-Oxoglutarate ↔ CO2 + 3-Carboxy-1-hydroxypropyl-ThPP	R00621
22	2-Phospho-D-glycerate ↔ H2O + Phosphoenolpyruvate	R00658
23	NAD+ + (S)-Lactate ↔ Pyruvate + NADH + H+	R00703
24	NAD+ + Ethanol ↔ NADH + H+ + Acetaldehyde	R00754
25	D-Glyceraldehyde 3-phosphate ↔ Glycerone phosphate	R01015
26	ATP + D-Ribose 5-phosphate ↔ AMP + 5-Phospho-alpha-D-ribose 1-diphosphate	R01049
27	D-Ribose 5-phosphate ↔ D-Ribulose 5-phosphate	R01056
28	Orthophosphate + NAD+ + D-Glyceraldehyde 3-phosphate ↔ NADH + H+ + 3-Phospho-D-glyceroyl phosphate	R01061
29	beta-D-Fructose 1,6-bisphosphate ↔ D-Glyceraldehyde 3-phosphate + Glycerone phosphate	R01070
30	(S)-Malate ↔ H2O + Fumarate	R01082
31	Citrate ↔ Isocitrate	R01324
32	Citrate ↔ H2O + cis-Aconitate	R01325
33	ATP + 3-Phospho-D-glycerate ↔ ADP + 3-Phospho-D-glyceroyl phosphate	R01512
34	2-Phospho-D-glycerate ↔ 3-Phospho-D-glycerate	R01518
35	NADP+ + 6-Phospho-D-gluconate ↔ CO2 + H+ + NADPH + D-Ribulose 5-phosphate	R01528
36	D-Ribulose 5-phosphate ↔ D-Xylulose 5-phosphate	R01529
37	ATP + beta-D-Glucose ↔ ADP + beta-D-Glucose 6-phosphate	R01600
38	D-Glyceraldehyde 3-phosphate + Sedoheptulose 7-phosphate ↔ D-Ribose 5-phosphate + D-Xylulose 5-phosphate	R01641
39	NAD+ + Dihydrolipoamide ↔ NADH + H+ + Lipoamide	R01698
40	D-Glyceraldehyde 3-phosphate + Sedoheptulose 7-phosphate ↔ D-Erythrose 4-phosphate + beta-D-Fructose 6-phosphate	R01827
41	D-Glyceraldehyde 3-phosphate + beta-D-Fructose 6-phosphate ↔ D-Xylulose 5-phosphate + D-Erythrose 4-phosphate	R01830
42	NADP+ + Isocitrate ↔ H+ + NADPH + Oxalosuccinate	R01899
43	Isocitrate ↔ H2O + cis-Aconitate	R01900
44	H2O + D-Glucono-1,5-lactone 6-phosphate ↔ 6-Phospho-D-gluconate	R02035
45	6-Phospho-D-gluconate ↔ H2O + 2-Dehydro-3-deoxy-6-phospho-D-gluconate	R02036
46	Acetyl-CoA + Enzyme N6-(dihydrolipoyl)lysine ↔ CoA + [Dihydrolipoyllysine-residue acetyltransferase]S-acetyldihydrolipoyllysine	R02569
47	Succinyl-CoA + Enzyme N6-(dihydrolipoyl)lysine ↔ CoA + [Dihydrolipoyllysine-residue succinyltransferase]S-succinyldihydrolipoyllysine	R02570
48	NADP+ + beta-D-Glucose 6-phosphate ↔ H+ + NADPH + D-Glucono-1,5-lactone 6-phosphate	R02736
49	Pyruvate + H2O + Ferricytochrome b1 ↔ CO2 + Acetate + Ferrocytochrome b1	R03145
50	2-(alpha-Hydroxyethyl)thiamine diphosphate + Enzyme N6-(lipoyl)lysine ↔ Thiamin diphosphate + [Dihydrolipoyllysine-residue acetyltransferase]S-acetyldihydrolipoyllysine	R03270

51	3-Carboxy-1-hydroxypropyl-ThPP + Enzyme N6-(lipoyl)lysine ↔ Thiamin diphosphate + [Dihydrolipoyllysine-residue succinyltransferase]S-succinyldihydrolipoyllysine	R03316
52	beta-D-Glucose 6-phosphate ↔ beta-D-Fructose 6-phosphate	R03321
53	ATP + beta-D-Fructose 6-phosphate ↔ ADP + beta-D-Fructose 1,6-bisphosphate	R04779
54	H2O + beta-D-Fructose 1,6-bisphosphate ↔ Orthophosphate + beta-D-Fructose 6-phosphate	R04780
55	2-Dehydro-3-deoxy-6-phospho-D-gluconate ↔ Pyruvate + D-Glyceraldehyde 3-phosphate	R05605
56	ATP + AMP ↔ 2 ADP	R00127
57	H2O + sn-Glycerol 3-phosphate ↔ Orthophosphate + Glycerol	R00841
58	NAD+ + sn-Glycerol 3-phosphate ↔ NADH + H+ + Glycerone phosphate	R00842
59	GlycerolExt ↔ Glycerol	–
60	beta-D-GlucoseExt ↔ beta-D-Glucose	–
61	NADPH ↔ NADP+	–
62	ADP + GTP ↔ ATP + GDP	–
63	1.50 ADP + Reduced acceptor + 0.50 O2 ↔ 1.50 ATP + H2O + Acceptor	–
64	1.50 ADP + NADH + 0.50 O2 ↔ 1.50 ATP + H2O + NAD+	–
65	ATP ↔ ADP	–
66	0.03 Pyruvate + 0.01 CO2 + 0.81 ATP + 0.02 Phosphoenolpyruvate + 0.03 2-Oxoglutarate + 0.02 Oxaloacetate + 0.08 NAD+ + 0.07 Acetyl-CoA + 0.02 D-Glyceraldehyde-3-phosphate + 0.01 D-Ribose-5-phosphate + 0.02 beta-D-Fructose-6-phosphate + 0.01 D-Erythrose-4-phosphate + 0.36 NADPH + 0.03 beta-D-Glucose-6-phosphate + 0.01O2 ↔ 0.81 ADP + 0.08 NADH + 0.36 NADP+ + 0.07 CoA + Biomass	–

2 Simulated experiments

The following tables describe the settings used to generate the simulated experimental data. Tables show the concentrations of enzymes and external metabolites. The initial concentrations of the internal metabolites are considered as model parameters and as such they are shown in subsection 3.1.

2.1 Enzyme concentrations

Enzyme concentrations of the 66 reactions (rows 1–66) for 10 different simulations (columns A–J).

	A	B	C	D	E	F	G	H	I	J
1	3.0875	0.2739	0.5982	1.7497	0.7275	0.6666	0.1524	1.1433	5.3308	1.5214
2	0.0890	0.4005	1.4877	11.531	0.2170	0.1246	0.5204	0.6961	1.5471	0.1025
3	3.1348	1.1679	0.8621	0.7084	0.3488	0.2579	5.1479	1.0879	0.7495	0.8566
4	0.2038	0.1511	3.9526	0.4596	2.7218	0.1538	1.7760	1.8232	0.1549	0.5320
5	0.1202	1.6718	2.0982	1.0439	1.1664	5.3069	2.8318	0.4523	0.1107	0.7366
6	7.3445	0.2446	0.9336	0.7093	7.8037	0.2818	3.6292	0.1212	0.6462	0.6554
7	1.6324	4.2880	1.6350	1.4058	2.5254	3.6045	3.1117	0.7821	0.9741	7.6021
8	4.7007	6.3016	0.2002	0.2325	0.5251	6.7944	0.7372	0.4253	0.2617	0.5846
9	1.3466	0.7040	0.3522	0.9088	1.2156	1.6126	0.1928	1.3778	2.3838	0.8095
10	0.7951	1.9425	0.9889	0.1790	3.1284	5.1847	20.077	0.3037	1.2074	0.3442
11	4.2332	0.3994	4.0194	0.6345	1.3374	1.6929	0.8228	0.9206	0.3802	0.6245
12	1.6381	0.1694	0.7424	1.1540	0.2271	0.4320	1.4213	2.6069	0.7038	0.9801
13	0.9496	1.2695	0.7068	0.3205	12.857	0.6034	0.0596	1.5563	0.1371	1.2308
14	0.8187	0.2353	2.4814	0.9752	13.180	0.1634	0.4775	0.1793	10.067	6.4490
15	0.0905	1.4083	1.4954	1.1593	0.5341	0.0761	2.0545	0.9043	1.9932	1.2475

16	3.7364	0.3941	1.3713	6.9204	2.2439	5.9189	11.871	0.3142	0.3430	0.4085
17	1.3381	2.3467	1.0033	0.7608	0.1484	0.7861	0.8945	0.6361	0.9472	1.6499
18	1.4795	1.2097	0.4058	2.7596	0.3065	1.4885	0.3281	0.3401	0.8351	5.2381
19	0.1272	2.9968	0.5505	2.2705	1.2470	0.2307	0.8688	0.1688	0.3688	0.2925
20	0.0300	1.1208	0.2449	0.3263	2.4677	0.1055	1.7529	1.3829	1.8347	0.4300
21	1.1142	0.7481	1.9434	1.4744	0.4370	1.4207	0.1522	3.3857	0.8734	0.9737
22	0.4321	0.3793	1.7703	0.7013	0.4918	1.3279	9.9392	10.330	0.5732	1.2926
23	0.3947	0.2633	0.1448	1.6806	0.5979	0.3379	1.6473	3.4026	2.1809	0.1445
24	0.2079	0.6627	1.8605	0.2201	3.8311	0.2913	0.4724	0.5046	0.3089	1.2896
25	1.5509	0.7983	2.5075	4.9962	0.1371	0.2184	0.2905	2.6145	0.3819	0.3801
26	0.4529	3.8861	0.1530	0.3295	6.8026	0.4514	1.6680	1.3055	0.6930	1.2161
27	0.1465	0.1397	0.5490	1.6581	3.5314	0.7445	1.1230	0.2783	2.6635	0.2350
28	0.6253	2.4897	0.4750	0.4313	1.7390	0.2069	2.3272	1.0373	0.9415	0.5994
29	9.6191	0.5880	0.9980	1.4075	0.1313	0.1992	0.5604	0.0415	0.6209	4.1854
30	1.8957	0.5575	0.8021	1.5791	0.2785	0.1680	4.9614	1.3807	1.2392	0.4094
31	0.6035	21.017	1.3825	0.2553	0.9772	0.4759	0.9920	1.1260	0.4966	0.1924
32	1.2526	3.7166	2.5090	0.7184	1.7839	0.9100	7.5187	4.4889	4.4860	4.1093
33	0.1310	3.1731	1.8935	13.457	9.7007	0.7056	23.503	0.9200	0.1286	3.2092
34	1.3318	1.0926	1.0727	0.3797	1.2587	1.2341	2.5905	3.4758	1.7430	0.2516
35	0.5672	0.3978	0.4294	0.5291	0.2239	0.2584	0.1113	4.1907	0.8444	1.8062
36	0.5467	0.9104	0.4944	0.5659	0.1985	0.3831	1.3682	1.1491	0.4008	0.2727
37	0.1112	0.2437	12.893	0.2899	0.2113	0.9315	1.8713	0.6333	0.3615	0.4777
38	0.6764	1.2718	0.5254	1.6309	2.1524	0.8038	2.4268	1.2273	0.2800	0.0910
39	1.1625	1.2013	0.2014	0.6262	0.5912	11.947	1.2128	0.2442	1.6866	1.0453
40	0.9153	0.4334	0.0400	3.6270	1.2831	0.4198	0.6464	0.6038	12.113	1.0401
41	0.5925	5.1677	0.6865	0.1148	1.6959	2.2776	0.0946	1.2831	1.3299	0.4497
42	0.3504	1.2708	8.9793	4.3656	0.4285	0.6426	0.7298	2.0447	0.5752	1.2182
43	0.5425	2.5638	0.2253	4.3816	11.639	47.280	0.3806	3.8254	0.4025	4.8668
44	17.450	1.4986	0.3930	0.5117	0.3378	2.0758	0.9115	0.0428	0.4311	0.7099
45	0.4915	0.3540	1.1096	3.5515	6.3600	0.6724	0.2085	5.0792	2.9425	1.9027
46	1.7781	1.4761	2.1954	2.1080	3.6841	0.7798	1.0638	0.5869	1.1892	0.1696
47	1.6520	1.8145	0.3227	1.4578	2.6818	0.8041	1.5178	0.8132	1.0346	11.833
48	1.1191	3.1688	0.5745	0.7739	8.2659	1.0534	0.3098	6.5973	2.4588	1.9217
49	0.8479	0.2453	3.4652	0.0520	0.6417	1.7446	0.6162	2.0513	0.9712	3.2396
50	0.8972	0.1259	0.4164	0.2078	17.780	9.8072	0.5572	4.5536	2.0345	4.8546
51	0.2448	1.6388	3.7811	2.1258	12.182	3.4704	0.6360	1.4527	2.9200	3.2565
52	3.2902	2.2980	4.4756	0.8509	1.2317	0.7095	1.0338	3.4064	0.2843	1.7534
53	0.1090	0.5782	0.9718	0.0905	0.1723	0.2730	0.1625	2.6562	5.5085	0.5524
54	0.8654	2.5305	0.3005	0.3698	0.4390	2.0490	5.7470	0.7520	0.3201	0.2285
55	8.8759	0.7535	0.3072	0.3375	6.8534	3.0136	0.1134	0.5114	0.8068	3.5466
56	0.2341	11.354	0.9368	0.2673	0.0376	0.8412	1.7153	2.8652	12.080	2.0061
57	0.3139	2.4511	2.4692	0.9122	0.9404	1.6559	1.9806	3.9128	0.1055	0.5196
58	0.3392	0.9010	0.4256	2.9783	0.3243	1.8614	2.1732	2.3273	0.7798	1.1173
59	0.5872	0.2499	4.4897	4.4103	0.9399	0.3646	4.9066	0.7973	1.4999	1.6353
60	0.7934	2.8611	0.4748	2.4916	16.834	1.4177	0.6320	9.5744	12.095	3.8821
61	0.5507	0.2051	1.5890	1.6063	0.8532	4.5125	0.3885	0.8369	2.7221	0.7024
62	0.5798	0.3722	2.3167	0.8554	0.0858	1.2938	0.5463	1.4489	1.9338	0.3883
63	3.7908	0.2563	0.1441	0.1234	1.5102	1.8544	2.8162	0.4481	0.9680	0.2605
64	2.4924	0.2457	8.9572	0.5830	2.1022	0.6667	0.8604	0.4823	4.6196	2.0119
65	6.4737	1.8841	0.4138	3.7397	0.3199	0.4996	23.413	1.1499	0.5086	0.8072
66	1.8798	5.1262	0.3837	1.0207	0.2839	0.3192	1.2828	0.5729	4.8585	2.4548

2.2 External metabolites

Initial concentrations of the 25 external metabolites (rows 1–25) for 10 different simulations (columns A–J).

	A	B	C	D	E	F	G	H	I	J
1	0.7745	0.7394	1.0491	0.0996	1.9480	5.6463	0.2772	1.7261	0.2784	2.3886
2	0.5511	0.6154	5.0607	0.1721	0.4141	0.0557	0.7802	8.4745	3.5364	0.3204
3	0.4482	1.6624	0.8909	1.0199	0.4503	5.3050	0.0412	6.3607	0.3643	3.9547
4	0.5596	2.3263	0.3290	6.8223	0.4528	0.0753	0.1034	0.8971	1.1018	1.4192
5	1.2030	1.4850	0.3539	0.2038	8.0203	2.3646	2.9425	3.5347	8.5326	4.3255
6	0.6513	8.1288	1.1018	2.3705	0.1292	6.2188	2.2313	1.1621	0.5363	0.6246
7	0.9699	1.2813	1.0258	0.2957	0.9561	2.3214	0.7129	0.5842	2.7016	1.6770
8	1.6515	0.8312	0.5238	0.2369	6.0328	0.5382	5.6048	1.0170	2.8337	1.2144
9	1.5883	3.2292	2.4515	5.7710	0.5202	1.3925	1.2612	4.6925	0.3053	1.1532
10	4.6273	2.8738	0.6056	0.4238	0.6896	0.8915	0.6964	0.3247	0.1282	7.3826
11	0.9425	1.8451	4.4263	0.5952	3.9774	2.0241	0.7980	0.5187	0.6970	0.7472
12	0.7214	7.3474	1.0752	0.6455	1.0549	0.1312	0.1494	1.8013	2.2059	0.6207
13	1.0724	0.4471	2.1867	0.8116	0.4246	1.1753	0.7623	1.5997	1.1124	0.7388
14	0.5307	0.7315	1.2261	2.3500	54.8789	1.9077	5.2710	0.4195	1.3335	1.8440
15	1.1640	7.4690	0.3555	0.8749	0.2238	2.1761	0.6713	0.7924	0.0891	0.7769
16	0.3478	0.1809	0.1156	1.4206	0.3746	0.0868	0.4225	0.6652	0.1547	9.3976
17	3.7393	4.8668	1.1948	12.8239	0.6167	1.3059	2.9706	0.1896	0.1355	2.5697
18	1.0625	0.5394	0.5762	0.3746	1.0389	3.2549	0.5817	1.8060	4.5252	3.6219
19	0.7040	0.1426	0.3258	1.7245	7.4730	2.7653	0.9652	5.8586	0.2309	2.1693
20	0.3409	0.8182	7.5783	3.1022	0.5634	3.9735	2.9988	4.1127	1.2774	1.4260
21	0.2613	0.6800	2.9173	2.5854	6.1633	0.1827	0.2414	0.7114	0.9957	0.1590
22	0.1516	1.0048	2.4911	1.2493	0.8863	0.6485	9.1619	0.7161	0.6752	1.8700
23	4.9567	0.6589	0.5613	0.7280	1.8000	1.4837	0.6234	1.1266	15.4360	0.0937
24	4.3802	2.0541	0.8464	0.0255	0.6818	1.0299	0.1980	0.5735	0.1774	0.7604
25	2.9196	1.5713	3.1989	0.2758	2.0177	2.8235	4.7079	1.9119	7.5151	0.7773

3 Model parameters

The following tables show the nominal values of the model parameters, $\mathbf{p}$. The upper and lower bounds used in the optimizations are $\mathbf{p}^L = 0.1 \cdot \mathbf{p}$; $\mathbf{p}^U = 10 \cdot \mathbf{p}$.

3.1 Model parameters (1–490)

Initial concentrations of the 49 internal metabolites (rows 1–49) for 10 different simulations (columns A–J).

	A	B	C	D	E	F	G	H	I	J
1	1.6315	2.4785	3.7780	0.4496	0.4091	0.1761	5.9890	5.9215	11.662	0.1757
2	2.7140	2.6434	0.5199	0.1728	2.9693	90.590	2.7203	2.6895	1.2851	4.7203
3	2.1322	1.4996	1.0136	1.8411	10.213	1.7352	2.0359	0.7954	1.6666	0.4591
4	0.8937	1.1100	1.9273	3.5181	2.5185	2.2196	1.9986	2.5362	0.6982	0.2172
5	2.3686	3.0015	4.3752	0.0533	0.6958	0.8263	7.3733	4.6877	17.849	0.2510
6	1.8981	1.2147	0.8308	1.8434	9.7131	1.7932	1.8432	1.2588	0.9744	0.4801
7	1.0389	1.0152	11.3564	0.0241	0.7202	0.0786	7.5634	36.941	60.798	0.1786
8	2.1417	1.5188	2.8933	0.0812	2.4580	0.1925	1.9028	3.4990	0.8607	1.7280

9	0.4859	0.9458	3.9599	0.6926	1.0358	0.1502	0.2775	0.9440	0.1184	1.9251
10	0.2362	1.0973	1.2226	1.3080	1.4345	0.6451	3.5632	1.4020	9.7109	0.6807
11	0.2194	1.7625	1.3614	1.4054	1.2143	0.6782	2.1444	1.3842	10.456	0.6793
12	0.5981	0.7867	1.1842	1.2808	22.166	0.6339	4.2155	1.5988	1.8486	2.3156
13	0.9790	2.6470	2.0172	5.0001	1.5082	0.1430	1.0714	2.7287	1.0570	1.7934
14	2.3927	1.0984	9.8048	0.0157	0.4895	60.235	1.1082	31.570	15.438	2.2718
15	1.3188	0.9843	51.2142	0.0049	0.1924	2.7892	1.1212	239.45	42.008	0.6025
16	1.8284	1.1018	15.0789	0.0022	0.2756	0.1852	6.2791	62.169	154.99	0.1283
17	2.7630	2.1950	32.9400	0.0105	0.1514	0.2541	7.4662	219.76	87.347	0.1754
18	0.6057	3.8270	2.1308	0.2905	1.1677	1.5440	0.3097	30.684	2.1741	0.8016
19	1.0773	2.4446	8.5334	0.0155	4.6614	77.459	1.5786	1.7970	5.7148	0.6912
20	0.8026	0.5148	4.2872	0.0880	0.9459	0.3797	3.3797	0.9206	1.4737	0.1112
21	2.3116	0.7375	11.5715	0.0243	1.7900	0.2362	140.42	5.7954	119.60	0.0577
22	0.1919	2.3328	0.8431	0.9663	0.5333	2.0963	0.0784	4.3032	0.0858	1.4194
23	2.9102	0.5761	21.1421	0.0321	0.1541	0.0810	25.151	6.4350	50.753	0.1245
24	2.9136	2.3477	33.6017	0.0083	0.1745	0.3200	7.3817	230.03	111.54	0.1963
25	3.5964	1.2630	2.1128	0.0023	1.7267	3946.9	1.1245	7.4587	1.9940	22.301
26	0.9880	5.6923	3.3891	0.0638	0.1854	4.0265	0.3197	27.109	5.4948	0.8909
27	0.7761	2.0027	1.7172	2.5115	0.4809	1.7897	7.1452	1.0137	1.5360	0.5107
28	0.6861	1.8695	1.4931	2.2366	0.4148	0.8091	9.7066	0.8292	1.6894	0.4397
29	0.7329	5.3734	0.9901	1.7104	0.0685	2.2726	0.7439	2.2979	0.3935	0.3890
30	1.1864	3.9814	1.0819	1.8254	0.0963	2.3375	0.8166	2.3654	0.4654	0.4823
31	1.0672	7.3691	3.1381	0.0829	0.1523	3.4982	0.3597	9.0385	5.2529	0.6843
32	0.4712	3.4146	2.2240	4.8405	0.2356	0.7694	60.337	1.0127	2.5842	0.1968
33	6.3949	1.3720	38.3924	0.0085	0.3687	0.0582	188.15	35.635	251.36	0.0471
34	0.9476	5.7340	3.0628	0.0835	0.1548	3.8363	0.3277	24.913	5.2086	0.7733
35	0.5916	5.8581	3.9803	0.6792	0.0789	2.8772	0.0339	21.597	4.7214	0.2500
36	1.4191	3.8735	1.1329	1.7126	0.4333	1.8551	0.9185	2.3006	0.8541	0.4761
37	4.6603	0.6647	0.6049	0.7191	1.7607	1.4381	0.6270	1.1226	15.181	0.0926
38	1.9608	5.3788	0.7094	0.8306	0.5373	1.1760	0.6756	2.6324	25.930	0.1947
39	1.8533	9.4687	0.6443	1.0839	0.1595	1.8500	0.1217	4.4747	252.96	0.1168
40	0.8775	2.8818	1.0935	2.7888	0.3611	1.5530	1.8874	1.3309	25.124	0.2557
41	1.7668	5.1042	0.6568	0.8941	0.4047	1.4073	0.5808	2.4995	14.701	0.1868
42	1.2882	3.4873	4.1340	0.9091	0.1064	0.6211	0.4701	2.3961	8.2806	0.0706
43	1.2168	4.4393	4.9899	0.7083	0.1855	0.3482	36.887	3.5816	14.642	0.0802
44	1.2536	0.6790	0.2849	0.3310	8.7366	2.9087	2.4306	1.5562	9.6378	0.5130
45	1.1507	1.0314	5.3716	0.0225	5.0403	113.08	1.2064	3.7235	7.9940	1.4485
46	0.2293	1.1762	0.8998	6.8808	0.8969	0.0391	9.9605	0.1405	0.8810	0.6357
47	0.1487	0.8628	2.3926	1.1775	0.8574	0.7683	8.5788	0.8151	0.6362	1.7889
48	3.5140	0.5154	0.7468	4.3293	4.2228	1.4059	0.6129	0.4422	1.8534	1.1753
49	3.4268	0.4573	0.6705	4.7484	4.3995	1.5916	0.6078	0.7666	1.1870	1.3464

3.2 Model parameters (491–786)

Activation (k_A), Inhibition (k_I), and Michaelis-Menten (k_M) constants. The third column shows the corresponding reaction and metabolite number.

Param. number	Type	(Reaction, Metabolite)	Param. value

491	k_A	(3,7)	0.5454
492	k_A	(53,7)	1.0568
493	k_A	(53,10)	0.9438
494	k_A	(53,72)	2.2919
495	k_I	(54,5)	1.4613
496	k_I	(54,7)	0.9920
497	k_I	(53,8)	1.2174
498	k_I	(54,8)	1.0576
499	k_I	(54,9)	1.0079
500	k_I	(54,10)	0.8153
501	k_I	(15,16)	1.6785
502	k_I	(48,16)	0.4463
503	k_I	(15,25)	0.3441
504	k_M	(1,1)	1.6597
505	k_M	(2,1)	1.4926
506	k_M	(3,1)	1.0283
507	k_M	(4,1)	1.5989
508	k_M	(5,1)	1.4834
509	k_M	(6,1)	0.8376
510	k_M	(23,1)	2.1174
511	k_M	(49,1)	0.4301
512	k_M	(55,1)	1.0933
513	k_M	(66,1)	1.1742
514	k_M	(1,2)	0.5036
515	k_M	(21,2)	1.3999
516	k_M	(50,2)	1.2932
517	k_M	(51,2)	1.6567
518	k_M	(1,3)	2.2819
519	k_M	(50,3)	0.5713
520	k_M	(1,4)	0.9922
521	k_M	(5,4)	0.8328
522	k_M	(6,4)	0.3156
523	k_M	(11,4)	0.5207
524	k_M	(13,4)	0.4927
525	k_M	(21,4)	1.0507
526	k_M	(35,4)	1.5372
527	k_M	(49,4)	0.8264
528	k_M	(66,4)	1.6215
529	k_M	(2,5)	1.5544
530	k_M	(3,5)	2.4796
531	k_M	(9,5)	1.0844
532	k_M	(12,5)	0.4728
533	k_M	(13,5)	0.9675
534	k_M	(17,5)	0.9754
535	k_M	(26,5)	1.0290
536	k_M	(33,5)	2.5306
537	k_M	(37,5)	3.0732
538	k_M	(53,5)	1.1723
539	k_M	(56,5)	0.7132
540	k_M	(62,5)	1.3087

541	k_M	(63,5)	1.3132
542	k_M	(64,5)	0.3062
543	k_M	(65,5)	1.1711
544	k_M	(66,5)	1.6084
545	k_M	(2,6)	0.4991
546	k_M	(10,6)	3.1041
547	k_M	(15,6)	1.8105
548	k_M	(19,6)	2.8992
549	k_M	(22,6)	1.3851
550	k_M	(30,6)	1.3111
551	k_M	(32,6)	0.7547
552	k_M	(43,6)	3.6565
553	k_M	(44,6)	0.4326
554	k_M	(45,6)	0.5813
555	k_M	(49,6)	0.5126
556	k_M	(54,6)	0.8903
557	k_M	(57,6)	0.7065
558	k_M	(63,6)	0.9037
559	k_M	(64,6)	1.6754
560	k_M	(2,7)	0.9490
561	k_M	(9,7)	0.6322
562	k_M	(26,7)	0.5684
563	k_M	(56,7)	1.6856
564	k_M	(2,8)	1.0311
565	k_M	(3,8)	0.8043
566	k_M	(13,8)	0.6735
567	k_M	(22,8)	0.4166
568	k_M	(66,8)	1.2224
569	k_M	(2,9)	1.1866
570	k_M	(8,9)	2.9321
571	k_M	(17,9)	0.5166
572	k_M	(28,9)	1.0571
573	k_M	(54,9)	0.9131
574	k_M	(57,9)	0.9421
575	k_M	(3,10)	2.9105
576	k_M	(12,10)	1.9695
577	k_M	(13,10)	1.0128
578	k_M	(17,10)	0.9242
579	k_M	(33,10)	1.1009
580	k_M	(37,10)	2.2203
581	k_M	(53,10)	0.6965
582	k_M	(56,10)	1.7502
583	k_M	(62,10)	2.0461
584	k_M	(63,10)	0.4823
585	k_M	(64,10)	1.2853
586	k_M	(65,10)	0.9748
587	k_M	(66,10)	0.4270
588	k_M	(4,11)	0.2581
589	k_M	(7,11)	0.3028
590	k_M	(8,11)	2.4001
591	k_M	(9,11)	0.6596

592	k_M	(15,11)	0.9815
593	k_M	(19,11)	1.6880
594	k_M	(46,11)	1.8131
595	k_M	(66,11)	0.8165
596	k_M	(4,12)	0.3561
597	k_M	(4,13)	1.1955
598	k_M	(7,13)	1.2649
599	k_M	(8,13)	1.2226
600	k_M	(9,13)	1.5826
601	k_M	(15,13)	2.5000
602	k_M	(17,13)	2.4916
603	k_M	(19,13)	0.3712
604	k_M	(46,13)	1.7825
605	k_M	(47,13)	0.2121
606	k_M	(66,13)	1.4793
607	k_M	(5,14)	0.4954
608	k_M	(6,14)	0.7861
609	k_M	(14,14)	0.5955
610	k_M	(19,14)	1.0791
611	k_M	(30,14)	1.8813
612	k_M	(5,15)	1.1597
613	k_M	(7,15)	1.0460
614	k_M	(10,15)	1.2548
615	k_M	(14,15)	0.5972
616	k_M	(23,15)	0.6909
617	k_M	(24,15)	0.5355
618	k_M	(28,15)	3.0387
619	k_M	(39,15)	1.4974
620	k_M	(58,15)	1.8680
621	k_M	(64,15)	0.7717
622	k_M	(66,15)	0.8953
623	k_M	(5,16)	1.5788
624	k_M	(7,16)	0.1225
625	k_M	(10,16)	1.8765
626	k_M	(14,16)	1.1161
627	k_M	(23,16)	2.0706
628	k_M	(24,16)	0.5876
629	k_M	(28,16)	0.5378
630	k_M	(39,16)	1.8108
631	k_M	(58,16)	0.3749
632	k_M	(64,16)	0.4807
633	k_M	(66,16)	0.4066
634	k_M	(5,17)	1.5788
635	k_M	(6,17)	0.1700
636	k_M	(7,17)	0.6902
637	k_M	(10,17)	0.4507
638	k_M	(14,17)	0.7765
639	k_M	(23,17)	0.5757
640	k_M	(24,17)	0.5738
641	k_M	(28,17)	1.8281
642	k_M	(35,17)	0.2841

643	k_M	(39,17)	1.4735
644	k_M	(42,17)	1.2184
645	k_M	(48,17)	0.3822
646	k_M	(58,17)	0.9463
647	k_M	(6,18)	0.6406
648	k_M	(35,18)	1.1325
649	k_M	(42,18)	0.6750
650	k_M	(48,18)	0.5410
651	k_M	(61,18)	0.6947
652	k_M	(66,18)	0.8948
653	k_M	(6,19)	0.4016
654	k_M	(35,19)	0.3420
655	k_M	(42,19)	1.3461
656	k_M	(48,19)	0.6620
657	k_M	(61,19)	0.3777
658	k_M	(66,19)	3.8272
659	k_M	(7,20)	2.3580
660	k_M	(24,20)	1.1756
661	k_M	(8,21)	4.1393
662	k_M	(12,21)	2.3599
663	k_M	(9,22)	1.1855
664	k_M	(12,22)	3.0182
665	k_M	(16,22)	1.0581
666	k_M	(49,22)	0.2327
667	k_M	(9,23)	1.2312
668	k_M	(10,24)	0.5297
669	k_M	(10,25)	2.1209
670	k_M	(11,25)	1.0996
671	k_M	(21,25)	2.3612
672	k_M	(66,25)	0.3067
673	k_M	(10,26)	0.8022
674	k_M	(11,27)	0.4459
675	k_M	(42,27)	4.3677
676	k_M	(13,28)	1.8255
677	k_M	(14,28)	0.5724
678	k_M	(15,28)	0.5935
679	k_M	(16,28)	0.5963
680	k_M	(66,28)	1.0077
681	k_M	(15,29)	1.1283
682	k_M	(16,29)	0.7150
683	k_M	(31,29)	1.1101
684	k_M	(32,29)	0.8219
685	k_M	(17,30)	0.5922
686	k_M	(18,30)	1.1154
687	k_M	(20,30)	1.1318
688	k_M	(17,31)	0.9193
689	k_M	(47,31)	0.8026
690	k_M	(18,32)	0.1628
691	k_M	(63,32)	1.7090
692	k_M	(18,33)	0.3208
693	k_M	(30,33)	0.5122

694	k_M	(18,34)	1.1154
695	k_M	(63,34)	2.6757
696	k_M	(19,35)	1.6653
697	k_M	(20,35)	0.6413
698	k_M	(20,36)	0.9638
699	k_M	(31,36)	0.6506
700	k_M	(42,36)	4.1343
701	k_M	(43,36)	1.0054
702	k_M	(21,37)	0.4806
703	k_M	(51,37)	1.2202
704	k_M	(22,38)	0.3819
705	k_M	(34,38)	1.4881
706	k_M	(23,39)	2.1333
707	k_M	(24,40)	0.9349
708	k_M	(25,41)	2.4053
709	k_M	(28,41)	0.7235
710	k_M	(29,41)	0.6160
711	k_M	(38,41)	0.5020
712	k_M	(40,41)	2.3416
713	k_M	(41,41)	1.0492
714	k_M	(55,41)	0.8979
715	k_M	(66,41)	0.5735
716	k_M	(25,42)	1.3945
717	k_M	(29,42)	1.0087
718	k_M	(58,42)	1.7969
719	k_M	(26,43)	0.9600
720	k_M	(27,43)	1.5149
721	k_M	(38,43)	4.3804
722	k_M	(66,43)	2.2841
723	k_M	(26,44)	1.4286
724	k_M	(27,45)	3.1591
725	k_M	(35,45)	0.3887
726	k_M	(36,45)	0.8627
727	k_M	(28,46)	1.0990
728	k_M	(33,46)	0.2615
729	k_M	(29,47)	1.3248
730	k_M	(53,47)	0.4676
731	k_M	(54,47)	1.8771
732	k_M	(32,48)	1.2395
733	k_M	(43,48)	1.5985
734	k_M	(33,49)	2.2014
735	k_M	(34,49)	0.5400
736	k_M	(35,50)	0.3851
737	k_M	(44,50)	1.2472
738	k_M	(45,50)	5.0491
739	k_M	(36,51)	1.3550
740	k_M	(38,51)	1.6822
741	k_M	(41,51)	1.8113
742	k_M	(37,52)	4.9762
743	k_M	(60,52)	0.6449
744	k_M	(37,53)	1.0313

745	k_M	(48,53)	1.6986
746	k_M	(52,53)	1.4272
747	k_M	(66,53)	0.2647
748	k_M	(38,54)	0.5338
749	k_M	(40,54)	1.4331
750	k_M	(39,55)	0.9637
751	k_M	(39,56)	1.5104
752	k_M	(40,57)	1.6346
753	k_M	(41,57)	1.0975
754	k_M	(66,57)	1.0220
755	k_M	(40,58)	0.6423
756	k_M	(41,58)	0.5379
757	k_M	(52,58)	1.9399
758	k_M	(53,58)	1.1711
759	k_M	(54,58)	0.9573
760	k_M	(66,58)	2.2425
761	k_M	(44,59)	0.5796
762	k_M	(48,59)	0.8518
763	k_M	(45,60)	1.1419
764	k_M	(55,60)	2.6337
765	k_M	(46,61)	0.9580
766	k_M	(47,61)	1.1886
767	k_M	(46,62)	0.5313
768	k_M	(50,62)	2.4624
769	k_M	(47,63)	1.0867
770	k_M	(51,63)	0.9561
771	k_M	(49,64)	0.4780
772	k_M	(49,65)	0.9961
773	k_M	(50,66)	2.0791
774	k_M	(51,66)	0.7623
775	k_M	(57,67)	0.9517
776	k_M	(58,67)	1.1778
777	k_M	(57,68)	1.7945
778	k_M	(59,68)	0.5279
779	k_M	(59,69)	0.8177
780	k_M	(60,70)	0.3235
781	k_M	(62,71)	2.5575
782	k_M	(62,72)	3.3308
783	k_M	(63,73)	0.9757
784	k_M	(64,73)	0.8116
785	k_M	(66,73)	0.3267
786	k_M	(66,74)	1.0427

3.3 Model parameters (787–918)

Velocity (k_v) and equilibrium (k_{eq}) constants of the reactions.

React. #	Param. number	k_v	Param. number	k_{eq}
1	787	9.5961	853	1.1286

2	788	8.5321	854	1.1162
3	789	10.1202	855	1.0648
4	790	10.2780	856	0.8955
5	791	8.9649	857	0.9556
6	792	11.2020	858	0.9616
7	793	11.2001	859	1.0159
8	794	9.9642	860	0.9720
9	795	10.3169	861	0.9668
10	796	10.1678	862	0.9486
11	797	9.8236	863	1.0023
12	798	10.7162	864	0.8708
13	799	9.4547	865	1.0364
14	800	12.3131	866	0.9818
15	801	9.8708	867	1.0272
16	802	10.1092	868	1.0450
17	803	11.0702	869	0.9180
18	804	10.0567	870	0.9287
19	805	9.9093	871	1.0254
20	806	9.2373	872	0.9844
21	807	10.2846	873	1.0647
22	808	8.8042	874	1.0809
23	809	10.7045	875	0.9075
24	810	11.6736	876	1.0346
25	811	9.3619	877	0.8805
26	812	10.8521	878	0.9942
27	813	11.2695	879	1.0361
28	814	8.5908	880	0.9361
29	815	8.7168	881	1.1336
30	816	10.5595	882	0.9857
31	817	9.6260	883	1.0758
32	818	10.6797	884	1.0408
33	819	10.8084	885	0.9733
34	820	10.7021	886	0.9582
35	821	11.3085	887	0.9904
36	822	10.6580	888	1.0672
37	823	11.2019	889	0.9340
38	824	8.9172	890	0.9725
39	825	9.9812	891	1.0054
40	826	9.8517	892	0.9412
41	827	8.5823	893	1.0731
42	828	10.2483	894	0.9312
43	829	9.0421	895	0.9675
44	830	11.4440	896	1.0236
45	831	9.2614	897	1.0066
46	832	10.5169	898	1.0308
47	833	10.2112	899	0.9768
48	834	9.1588	900	0.8980
49	835	8.1311	901	0.9043
50	836	9.9437	902	0.9261
51	837	9.0817	903	0.9268
52	838	10.6031	904	0.9191

53	839	10.4958	905	0.8727
54	840	11.7504	906	1.0444
55	841	10.5797	907	1.0430
56	842	9.4050	908	0.8695
57	843	10.3692	909	1.0016
58	844	9.0830	910	0.9498
59	845	9.9814	911	0.9576
60	846	9.9541	912	0.9833
61	847	10.0000	913	1.0204
62	848	9.7016	914	1.0954
63	849	11.1001	915	1.0991
64	850	8.3643	916	1.1617
65	851	10.4165	917	0.9243
66	852	10.8911	918	0.9329

4 Model outputs

The 115 model outputs are:

- Outputs 1–49: concentrations of the 49 internal metabolites listed in table 1.
- Outputs 50–115: concentrations of the 66 reaction fluxes listed in table 2.

5 Influence of the tuning parameters in the performance of CeSS

First we test what happens when the threads exchange information at different time intervals (τ). Figure 1 (top) shows the convergence curves, which plot the logarithm of the objective function value against the computation time. The performance of 10 individual, non cooperative threads is shown in black. It is compared to the performance of 10 cooperative threads which exchange information with different values of τ: 2.53 (approximately 12 hours in the hardware used, red curve), 2.70 (18 hours, green), 2.83 (24 hours, blue), 3.13 (48 hours, magenta), and 3.30 (72 hours, cyan). For this system, the performance of the cooperative algorithm is not significantly influenced by the choice of the time between information sharing: τ can range between 2.53 and 3.13 without affecting significantly the outcome of the optimization.
Next we test the influence of the other cooperative optimization parameter, the number of threads η. We fix $\tau = 12$ hours and compare the algorithm's performance for $\eta = 5$, 10, 20, and 30 threads. Results are shown in Fig. 1 (bottom); as expected, convergence improves when the number of threads increases. However, the improvement is not linear: the objective function value is very much reduced when changing from 5 to 10 threads, but it is only slightly reduced with 20 or 30 threads.

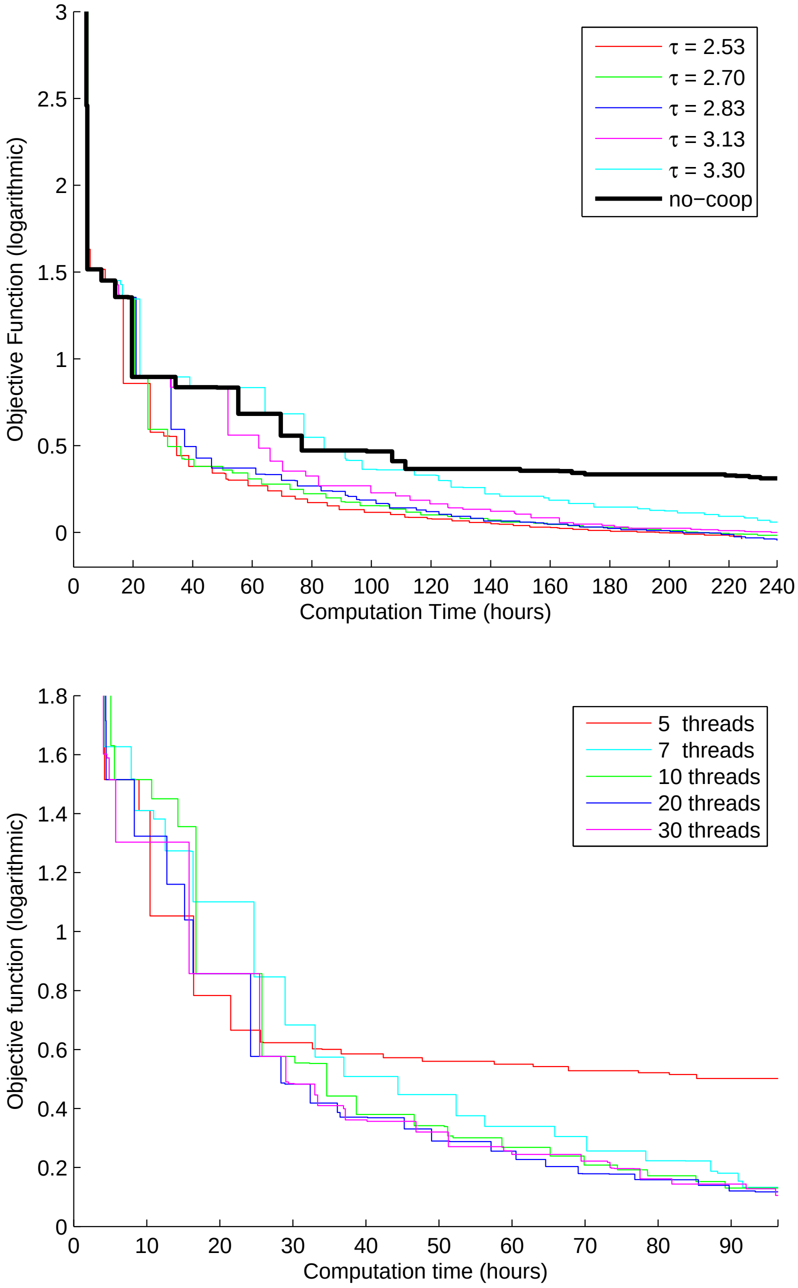

Figure 1: Convergence curves, Model 1. TOP: different τ, $\eta = 10$. BOTTOM: different η, $\tau = 2.53$.
