# Supplementary material for: A cooperative strategy for parameter estimation in large scale systems biology models
Source: BMC Syst Biol. 2012 Jun 22;6:75. doi: 10.1186/1752-0509-6-75 (PMC3512509; doi:10.1186/1752-0509-6-75)
Supplement: Additional file 3 — Model 2. The file includes information about the model structure and parameters, plots of the fits between the calibrated model and the simulated experimental data, and additional convergence curves showing the algorithm’s performance [ [18]]. [file 1752-0509-6-75-S3.pdf]

MODEL 2

1 Model Structure

Complete information about the model is available as the supplementary information of the article [1]; it can be retrieved from the open access webpage

`http://www.nature.com/msb/journal/v6/n1/suppinfo/msb201010_S1.html`

The model is also included in the BioModels Database: A Database of Annotated Published Models, where it can be accessed at `http://www.ebi.ac.uk/biomodels-main/BIOMD0000000244`.

It should be noted that there are some differences in parameter values between the original model and the BioModels version; however, these changes do not alter the simulation results. This fact shows that the model is unidentifiable.

2 Model parameters

The following tables show the nominal values of the model parameters, $\mathbf{p}$. The lower bounds used in the optimizations are $\mathbf{p}^L = 0.1 \cdot \mathbf{p}$; the upper bounds are $\mathbf{p}^U = 10 \cdot \mathbf{p}$ except for parameters with nominal values $p_i < 10^{-8}$, in which case the upper bounds are fixed to $\mathbf{p}^U = 10^{-7}$.

Param. number	Param. name	Description	Param. value
1	$p_{ENV,M_{ACT}}$	Molar mass of acetate	60.05 $g_{ACT} mol^{-1}$
2	$p_{ENV,M_{GLC}}$	Molar mass of glucose	180.156 $g_{GLC} mol^{-1}$
3	$p_{ENV,UC}$	Unit conversion	$9.5 \cdot 10^{-7} g_{DW} (\mu[OD])^{-1}$
4	$p_{AceA,K_{cat}}$	Specific activity	614 $\mu mol (g_{prots})^{-1}$
5	$p_{AceA,n}$	Number of subunits	4
6	$p_{AceA,L}$	Allosteric constant	$5.01 \cdot 10^4$
7	$p_{AceA,K_{ICT}}$	Affinity constant	0.022 $\mu mol g_{DW}^{-1}$
8	$p_{AceA,K_{PEP}}$	Affinity constant	0.055 $\mu mol g_{DW}^{-1}$
9	$p_{AceA,K_{PG3}}$	Affinity constant	0.72 $\mu mol g_{DW}^{-1}$
10	$p_{AceA,K_{AKG}}$	Affinity constant	0.827 $\mu mol g_{DW}^{-1}$
11	$p_{AceB,K_{cat}}$	Specific activity	47.8 $\mu mol (g_{Prot} s)^{-1}$
12	$p_{AceB,K_{GLX}}$	Affinity constant	0.95 $\mu mol g_{DW}^{-1}$
13	$p_{AceB,K_{ACoA}}$	Affinity constant	0.755 $\mu mol g_{DW}^{-1}$
14	$p_{AceB,K_{GLXACoA}}$	Affinity constant	0.719 $\mu mol g_{DW}^{-1}$
15	$p_{AceK,K_{cat,ki}}$	Specific activity	$3.4 \cdot 10^{12}$ s^{-1}
16	$p_{AceK,K_{cat,ph}}$	Specific activity	$1.7 \cdot 10^{9}$ s^{-1}
17	$p_{AceK,n}$	Number of subunits	2
18	$p_{AceK,L}$	Allosteric constant	$1 \cdot 10^8$
19	$p_{AceK,K_{Icd}}$	Affinity constant	0.043 $g_{prot} g_{DW}^{-1}$
20	$p_{AceK,K_{Icd-P}}$	Affinity constant	0.643 $g_{prot} g_{DW}^{-1}$
21	$p_{AceK,K_{PEP}}$	Affinity constant	0.539 $\mu mol g_{DW}^{-1}$
22	$p_{AceK,K_{PYR}}$	Affinity constant	0.038 $\mu mol g_{DW}^{-1}$

23	$p_{AceK,K_{OAA}}$	Affinity constant	$0.173\ \mu mol g_{DW}^{-1}$
24	$p_{AceK,K_{GLX}}$	Affinity constant	$0.866\ \mu mol g_{DW}^{-1}$
25	$p_{AceA,K_{AKG}}$	Affinity constant	$0.82\ \mu mol g_{DW}^{-1}$
26	$p_{AceK,K_{PG3}}$	Affinity constant	$1.57\ \mu mol g_{DW}^{-1}$
27	$p_{AceK,K_{ICT}}$	Affinity constant	$0.137\ \mu mol g_{DW}^{-1}$
28	$p_{Acoa2act,K_{cat}}$	Specific activity	$3079\ \mu mol (g_{Prot} s)^{-1}$
29	$p_{Acoa2act,n}$	Number of subunits	2
30	$p_{Acoa2act,L}$	Allosteric constant	$6.39 \cdot 10^5$
31	$p_{Acoa2act,K_{ACoA}}$	Affinity constant	$0.022\ \mu mol g_{DW}^{-1}$
32	$p_{Acoa2act,K_{PYR}}$	Affinity constant	$0.022\ \mu mol g_{DW}^{-1}$
33	$p_{Acs,k_{cat}}$	Specific activity	$1.0296 \cdot 10^4 \mu mol (g_{Prots})^{-1}$
34	$p_{Acs,K_{ACT}}$	Affinity constant	$10^{-3} g_{ACT} l^{-1}$
35	$p_{Akg2mal,k_{cat}}$	Specific activity	$1530\ \mu mol (g_{Prots})^{-1}$
36	$p_{Akg2mal,k_{AKG}}$	Affinity constant	$0.548\ \mu mol g_{DW}^{-1}$
37	$p_{CAMP_{degr},k_{cat}}$	Specific activity	$1 \cdot 10^3 \mu mol (g_{Prots})^{-1}$
38	$p_{CAMP_{degr},k_{cCAMP}}$	Affinity constant	$0.1\ \mu mol g_{DW}^{-1}$
39	$p_{Cya,k_{cat}}$	Specific activity	$993\ \mu mol (g_{Prots})^{-1}$
40	$p_{Cya,k_{EIIA-P}}$	Affinity constant	$1.7 \cdot 10^{-3} g_{Prot} g_{DW}^{-1}$
41	$p_{Emp,k_{cat},f}$	Spec. activ. forward react.	$1011\ \mu mol (g_{Prots})^{-1}$
42	$p_{Emp,k_{cat},r}$	Spec. activ. reverse react.	$857.4234\ \mu mol (g_{Prots})^{-1}$
43	$p_{Emp,K_{FBP}}$	Affinity constant	$5.92\ \mu mol g_{DW}^{-1}$
44	$p_{Emp,K_{PG3}}$	Affinity constant	$16.6\ \mu mol g_{DW}^{-1}$
45	$p_{Eno,k_{cat},f}$	Spec. activ. forward react.	$704.9945\ \mu mol (g_{Prots})^{-1}$
46	$p_{Eno,k_{cat},r}$	Spec. activ. reverse react.	$529.5067\ \mu mol (g_{Prots})^{-1}$
47	$p_{Eno,K_{PG3}}$	Affinity constant	$4.76\ \mu mol g_{DW}^{-1}$
48	$p_{Eno,K_{PEP}}$	Affinity constant	$1.11\ \mu mol g_{DW}^{-1}$
49	$p_{Fdp,k_{cat}}$	Specific activity	$404.2035\ \mu mol (g_{Prots})^{-1}$
50	$p_{Fdp,n}$	Number of subunits	4
51	$p_{Fdp,L}$	Allosteric constant	$4 \cdot 10^6$
52	$p_{Fdp,K_{FBP}}$	Affinity constant	$3 \cdot 10^{-3} \mu mol g_{DW}^{-1}$
53	$p_{Fdp,K_{PEP}}$	Affinity constant	$0.3 \mu mol g_{DW}^{-1}$
54	$p_{GltA,K_{cat}}$	Specific activity	$5.6761 \cdot 10^3 \mu mol (g_{Prots})^{-1}$
55	$p_{GltA,K_{OAA}}$	Affinity constant	$0.029 \mu mol g_{DW}^{-1}$
56	$p_{GltA,K_{ACoA}}$	Affinity constant	$0.212 \mu mol g_{DW}^{-1}$
57	$p_{GltA,K_{OAAACoA}}$	Affinity constant	$0.029 \mu mol g_{DW}^{-1}$
58	$p_{GltA,K_{AKG}}$	Affinity constant	$0.63 \mu mol g_{DW}^{-1}$
59	$p_{Icd,k_{cat}}$	Specific activity	$695 \mu mol (g_{Prots})^{-1}$
60	$p_{Icd,n}$	Number of subunits	2
61	$p_{Icd,L}$	Allosteric constant	127
62	$p_{Icd,K_{ICT}}$	Affinity constant	$1.6 \cdot 10^{-4} \mu mol g_{DW}^{-1}$
63	$p_{Icd,K_{PEP}}$	Affinity constant	$0.334 \mu mol g_{DW}^{-1}$
64	$p_{Mdh,k_{cat}}$	Specific activity	$5.4375 \cdot 10^3 \mu mol (g_{Prots})^{-1}$
65	$p_{Mdh,n}$	Hill coefficient	1.7
66	$p_{Mdh,k_{MAL}}$	Affinity constant	$10.1 \mu mol g_{DW}^{-1}$
67	$p_{MaeAB,k_{cat}}$	Specific activity	$1879\ \mu mol (g_{Prots})^{-1}$
68	$p_{MaeAB,n}$	Number of subunits	1.33
69	$p_{MaeAB,L}$	Allosteric constant	$1.04 \cdot 10^5$
70	$p_{MaeAB,K_{MAL}}$	Affinity constant	$6.24 \cdot 10^{-03} \mu mol g_{DW}^{-1}$
71	$p_{MaeAB,k_{ACoA}}$	Affinity constant	$3.64 \mu mol g_{DW}^{-1}$
72	$p_{MaeAB,k_{CAMP}}$	Affinity constant	$6.54 \mu mol g_{DW}^{-1}$

73	$p_{PckA,k_{cat}}$	Specific activity	$377.3427 \mu mol (g_{Prots})^{-1}$
74	$p_{PckA,k_{OAA}}$	Affinity constant	$0.184 \mu mol g_{DW}^{-1}$
75	$p_{PckA,k_{PEP}}$	Affinity constant	$1000 \mu mol g_{DW}^{-1}$
76	$p_{Pdh,k_{cat}}$	Specific activity	$5.4793 \cdot 10^3 \mu mol (g_{Prots})^{-1}$
77	$p_{Pdh,n}$	Number of subunits	2.65
78	$p_{Pdh,L}$	Allosteric constant	3.4
79	$p_{Pdh,K_{PYR}}$	Affinity constant	$0.128 \mu mol g_{DW}^{-1}$
80	$p_{Pdh,K_{I,PYR}}$	Affinity constant	$0.231 \mu mol g_{DW}^{-1}$
81	$p_{Pdh,K_{GLX}}$	Affinity constant	$0.218 \mu mol g_{DW}^{-1}$
82	$p_{PfkA,K_{cat}}$	Specific activity	$5.3932 \cdot 10^5 \mu mol (g_{Prots})^{-1}$
83	$p_{PfkA,n}$	Number of subunits	4
84	$p_{PfkA,L}$	Allosteric constant	$9.5 \cdot 10^7$
85	$p_{PfkA,K_{G6P}}$	Affinity constant	$0.022 \mu mol g_{DW}^{-1}$
86	$p_{PfkA,K_{PEP}}$	Affinity constant	$0.138 \mu mol g_{DW}^{-1}$
87	$p_{Ppc,K_{cat}}$	Specific activity	$1.4905 \cdot 10^4 \mu mol (g_{Prots})^{-1}$
88	$p_{Ppc,n}$	Number of subunits	3
89	$p_{Ppc,L}$	Allosteric constant	$5.2 \cdot 10^6$
90	$p_{Ppc,K_{PEP}}$	Affinity constant	$0.048 \mu mol g_{DW}^{-1}$
91	$p_{Ppc,K_{FBP}}$	Affinity constant	$0.408 \mu mol g_{DW}^{-1}$
92	$p_{PpsA,k_{cat}}$	Specific activity	$1.32 \mu mol (g_{Prots})^{-1}$
93	$p_{P_{psA},n}$	Number of subunits	2
94	$p_{P_{psA},L}$	Allosteric constant	$1.0 \cdot 10^{-79}$
95	$p_{P_{psA},K_{PYR}}$	Affinity constant	$1.77 \cdot 10^{-3} \mu mol g_{DW}^{-1}$
96	$p_{P_{psA},K_{PEP}}$	Affinity constant	$1.0 \cdot 10^{-3} \mu mol g_{DW}^{-1}$
97	$p_{P_{PykF},K_{cat}}$	Specific activity	$1.3735 \cdot 10^4 \mu mol (g_{Prots})^{-1}$
98	$p_{P_{PykF},n}$	Number of subunits	4
99	$p_{P_{PykF},L}$	Allosteric constant	$1.0 \cdot 10^4$
100	$p_{P_{PykF},K_{PEP}}$	Affinity constant	$5 \mu mol g_{DW}^{-1}$
101	$p_{P_{PykF},K_{FBP}}$	Affinity constant	$0.413 \mu mol g_{DW}^{-1}$
102	p_{P_{PTS},K_l}	Specific activity	$116 \mu mol (g_{Prots})^{-1}$
103	$p_{P_{PTS},K_{m_1}}$	Specific activity	$46.3 \mu mol (g_{Prots})^{-1}$
104	p_{P_{PTS},K_4}	Specific activity	$2520 \mu mol (g_{Prots})^{-1}$
105	$p_{P_{PTS},K_{EIIA}}$	Affinity constant	$8.5 \cdot 10^{-3} g_{Prot} g_{DW}^{-1}$
106	$p_{P_{PTS},K_{GLC}}$	Affinity constant	$1.2 \cdot 10^{-3} g_{GLC} l^{-1}$
107	$p_{P_{Cra},scale}$	Specific activity	$100 g_{Prot} (\mu mol s)^{-1}$
108	$p_{P_{Cra},K_{FBP}}$	Affinity constant	$1.36 \mu mol g_{DW}^{-1}$
109	$p_{P_{Cra},n}$	Hill coefficient	2
110	$p_{P_{Crp},scale}$	Specific activity	$1.0 \cdot 10^8 g_{Prot} (\mu mol s)^{-1}$
111	$p_{P_{Crp},K_{cAMP}}$	Affinity constant	$0.895 \mu mol g_{DW}^{-1}$
112	$p_{P_{Crp},n}$	Hill coefficient	1
113	$p_{P_{PdhR},scale}$	Specific activity	$100 g_{Prot} (\mu mol s)^{-1}$
114	$p_{P_{PdhR},K_{PYR}}$	Affinity constant	$0.164 \mu mol g_{DW}^{-1}$
115	$p_{P_{PdhR},n}$	Hill coefficient	1
116	$p_{aceBAK,v_{Cra},unbound}$	Basal expression rate	$1.9 \cdot 10^{-9} g_{Prot} (g_{DW} s)^{-1}$
117	$p_{aceBAK,v_{Cra},bound}$	Max. expression rate	$2.0 \cdot 10^{-6} g_{Prot} (g_{DW} s)^{-1}$
118	$p_{P_{aceBAK},K_{Cra}}$	Affinity constant	$3.65 \cdot 10^{-3} g_{Prot} g_{DW}^{-1}$
119	$p_{aceBAK,aceBfactor}$	Scaling factor	0.3
120	$p_{aceBAK,aceKfactor}$	Scaling factor	0.03
121	$p_{aceBAK,K_{DNA}}$	Affinity constant	2.19 [AU] g_{DW}^{-1}
122	$p_{aceBAK,K_{PYR}}$	Affinity constant	$0.897 \mu mol g_{DW}^{-1}$

123	$p_{aceBAK,K_{PYRprime}}$	Affinity constant	$3.01 \cdot 10^{-3} \mu mol g_{DW}^{-1}$
124	$p_{aceBAK,K_{GLX}}$	Affinity constant	$4.88 \cdot 10^{-3} \mu mol g_{DW}^{-1}$
125	$p_{aceBAK,L}$	Allosteric constant	923
126	$p_{aceBAK,K_{cat,IclR}}$	Specific activity	$9.3 \cdot 10^{-4} s^{-1}$
127	$p_{aceBAK,DNA}$	DNA concentration	1 [AU]g_{DW}^{-1}
128	$p_{aceBAK,v_{Crp,bound}}$	Basal expression rate	$2.3 \cdot 10^{-10} g_{Prot}(g_{DW}s)^{-1}$
129	$p_{aceBAK,v_{Crp,unbound}}$	Max. expression rate	$2.0 \cdot 10^{-8} g_{Prot}(g_{DW}s)^{-1}$
130	$p_{aceBAK,K_{Crp}}$	Affinity constant	$0.341\ g_{Prot}(g_{DW}s)^{-1}$
131	$p_{acs,v_{Crp,unbound}}$	Basal expression rate	$0\ g_{Prot}(g_{DW}s)^{-1}$
132	$p_{acs,v_{Crp,bound}}$	Max. expression rate	$3.9628 \cdot 10^{-8} g_{Prot}(g_{DW}s)^{-1}$
133	$p_{acs,n}$	Hill coefficient	2.31
134	$p_{acs,K_{Crp}}$	Affinity constant	$4.7 \cdot 10^{-3} g_{Prot} g_{DW}^{-1}$
135	$p_{akg2mal,v_{Crp,unbound}}$	Basal expression rate	$0\ g_{Prot}(g_{DW}s)^{-1}$
136	$p_{akg2mal,v_{Crp,bound}}$	Max. expression rate	$1.4 \cdot 10^{-6} g_{Prot}(g_{DW}s)^{-1}$
137	$p_{akg2mal,K_{Crp}}$	Affinity constant	$0.091\ g_{Prot} g_{DW}^{-1}$
138	$p_{akg2mal,n}$	Hill coefficient	0.74
139	$p_{emp,v_{Cra},unbound}$	Max. expression rate	$6.1319 \cdot 10^{-7} g_{Prot}(g_{DW}s)^{-1}$
140	$p_{emp,v_{Cra},unbound}$	Basal expression rate	$0\ g_{Prot}(g_{DW}s)^{-1}$
141	$p_{emp,K_{Cra}}$	Affinity constant	$0.09\ g_{Prot} g_{DW}^{-1}$
142	$p_{emp,v_{Crp,unbound}}$	Basal expression rate	$0\ g_{Prot}(g_{DW}s)^{-1}$
143	$p_{emp,v_{Crp,bound}}$	Max. expression rate	$4.7 \cdot 10^{-7} g_{Prot}(g_{DW}s)^{-1}$
144	$p_{emp,K_{Crp}}$	Affinity constant	$0.012\ g_{Prot} g_{DW}^{-1}$
145	$p_{eno,v_{Cra,unbound}}$	Max. expression rate	$6.7036 \cdot 10^{-7} g_{Prot}(g_{DW}s)^{-1}$
146	$p_{eno,v_{Cra,bound}}$	Basal expression rate	$0\ g_{Prot}(g_{DW}s)^{-1}$
147	$p_{eno,K_{Cra}}$	Affinity constant	$0.016\ g_{Prot} g_{DW}^{-1}$
148	$p_{fdp,v_{Cra,unbound}}$	Basal expression rate	$0\ g_{Prot}(g_{DW}s)^{-1}$
149	$p_{fdp,v_{Cra,bound}}$	Max. expression rate	$2.1375 \cdot 10^{-8} g_{Prot}(g_{DW}s)^{-1}$
150	$p_{fdp,K_{Cra}}$	Affinity constant	$1.18 \cdot 10^{-3} g_{Prot} g_{DW}^{-1}$
151	$p_{gltA,v_{Crp,unbound}}$	Basal expression rate	$0\ g_{Prot}(g_{DW}s)^{-1}$
152	$p_{gltA,v_{Crp,bound}}$	Max. expression rate	$6.5401 \cdot 10^{-7} g_{Prot}(g_{DW}s)^{-1}$
153	$p_{gltA,K_{Crp}}$	Affinity constant	$0.04\ g_{Prot} g_{DW}^{-1}$
154	$p_{gltA,n}$	Hill coefficient	1.07
155	$p_{icd,v_{Cra,unbound}}$	Basal expression rate	$1.1 \cdot 10^{-7} g_{Prot}(g_{DW}s)^{-1}$
156	$p_{icd,v_{Cra,bound}}$	Max. expression rate	$8.5 \cdot 10^{-7} g_{Prot}(g_{DW}s)^{-1}$
157	$p_{icd,K_{Cra}}$	Affinity constant	$1.17 \cdot 10^{-3} g_{Prot} g_{DW}^{-1}$
158	$p_{mdh,v_{Crp,unbound}}$	Basal expression rate	$0\ g_{Prot}(g_{DW}s)^{-1}$
159	$p_{mdh,v_{Crp,bound}}$	Max. expression rate	$1.2937 \cdot 10^{-6} g_{Prot}(g_{DW}s)^{-1}$
160	$p_{mdh,K_{Crp}}$	Affinity constant	$0.06\ g_{Prot} g_{DW}^{-1}$
161	$p_{pckA,v_{Cra,unbound}}$	Basal expression rate	$0\ g_{Prot} g_{DW}^{-1}$
162	$p_{pckA,v_{Cra,bound}}$	Max. expression rate	$3.6770 \cdot 10^{-7} g_{Prot}(g_{DW}s)^{-1}$
163	$p_{pckA,K_{Cra}}$	Affinity constant	$5.35 \cdot 10^{-3} g_{Prot} g_{DW}^{-1}$
164	$p_{pdh,v_{PdhR,unbound}}$	Max. expression rate	$7.7463 \cdot 10^{-8} g_{Prot}(g_{DW}s)^{-1}$
165	$p_{pdh,v_{PdhR,bound}}$	Basal expression rate	$2.7973 \cdot 10^{-7} g_{Prot}(g_{DW}s)^{-1}$
166	$p_{pdh,K_{PdhR}}$	Affinity constant	$3.4 \cdot 10^{-3} g_{Prot} g_{DW}^{-1}$
167	$p_{pfkA,v_{Cra,unbound}}$	Max. expression rate	$1.3806 \cdot 10^{-6} g_{Prot}(g_{DW}s)^{-1}$
168	$p_{pfkA,v_{Cra,bound}}$	Basal expression rate	$1.1112 \cdot 10^{-8} g_{Prot}(g_{DW}s)^{-1}$
169	$p_{pfkA,K_{Cra}}$	Affinity constant	$6.3 \cdot 10^{-7} g_{Prot} g_{DW}^{-1}$
170	$p_{ppsA,v_{Cra,unbound}}$	Basal expression rate	$0\ g_{Prot}(g_{DW}s)^{-1}$
171	$p_{ppsA,v_{Cra,unbound}}$	Max. expression rate	$3.3 \cdot 10^{-6} g_{Prot}(g_{DW}s)^{-1}$
172	$p_{ppsA,K_{Cra}}$	Affinity constant	$0.017\ g_{Prot} g_{DW}^{-1}$

173	$p_{pykF,v_{Cra,unbound}}$	Max. expression rate	$1.6324 \cdot 10^{-7} g_{Prot}(g_{DW} s)^{-1}$
174	$p_{pykF,v_{Cra,bound}}$	Basal expression rate	$8.7901 \cdot 10^{-10} g_{Prot}(g_{DW} s)^{-1}$
175	$p_{pykF,K_{Cra}}$	Affinity constant	$2.3 \cdot 10^{-3} g_{Prot} g_{DW}^{-1}$
176	$p_{D,K_{degr}}$	Univ. prot. degr. rate	$2.8 \cdot 10^{-5}\ s^{-1}$
177	$p_{BM,K_{expr}}$	Gene expr. rate const.	$2.0 \cdot 10^{4}\ s$
178	$p_{BM,\mu_{ACT}}$	Growth rate on acetate	$5.6 \cdot 10^{-5}\ s^{-1}$
179	$p_{BM,\mu_{GLC}}$	Growth rate on glucose	$1.8 \cdot 10^{-4}\ s^{-1}$
180	$p_{BM,GLC_{ACoA}}$	1st order rate constant	$1.88\ s^{-1}$
181	$p_{BM,GLC_{AKG}}$	1st order rate constant	$0.978\ s^{-1}$
182	$p_{BM,GLC_{G6P}}$	1st order rate constant	$0.154\ s^{-1}$
183	$p_{BM,GLC_{OAA}}$	1st order rate constant	$6.4\ s^{-1}$
184	$p_{BM,GLC_{PEP}}$	1st order rate constant	$0.423\ s^{-1}$
185	$p_{BM,GLC_{PG3}}$	1st order rate constant	$0.049\ s^{-1}$
186	$p_{BM,GLC_{PYR}}$	1st order rate constant	$0.553\ s^{-1}$
187	$p_{BM,ACT_{ACoA}}$	1st order rate constant	$0.108\ s^{-1}$
188	$p_{BM,ACT_{AKG}}$	1st order rate constant	$0.056\ s^{-1}$
189	$p_{BM,ACT_{G6P}}$	1st order rate constant	$0.076\ s^{-1}$
190	$p_{BM,ACT_{OOA}}$	1st order rate constant	$1.43\ s^{-1}$
191	$p_{BM,ACT_{PEP}}$	1st order rate constant	$0.047\ s^{-1}$
192	$p_{BM,ACT_{PG3}}$	1st order rate constant	$0.066\ s^{-1}$
193	$p_{BM,ACT_{PYR}}$	1st order rate constant	$5.185\ s^{-1}$

3 Model outputs

The following table lists the system's outputs, which are 47 output dynamic state variables.

Number	Name	Description
1	xOD	Biomass concentration
2	xxACT	Extracellular acetate
3	xxGLC	Extracellular glucose
4	xxACoA	Acetyl-CoA
5	xxAKG	α-Ketoglutarate
6	xxcAMP	Cyclic AMP
7	xxFBP	Fructose-1,6-bisphosphate
8	xxG6P	Glucose-6-phosphate
9	xxGLX	Glyoxylate
10	xxICT	Isocitrate
11	xxMAL	Malate
12	xxOAA	Oxaloacetate
13	xxPEP	Phosphoenolpyruvate
14	xxPG3	3-Phosphoglycerate
15	xxPY	R Pyruvate
16	xxAceA	Isocitrate lyase
17	xxAceB	Malate synthase A
18	xxAceK	Isocitrate dehydrogenase phosphatase/kinase
19	xxAcoa2act	Enzyme for the reaction from ACoA to ACT
20	xxAcs	Acetyl-CoA synthetase
21	xxAkg2mal	Enzyme for the reaction from AKG to MAL
22	xxCAMPdegr	Degradation of cAMP
23	xxCya	Adenylate cyclase

24	xxEmp	Enzyme for the reversible reaction between FBP and PG3
25	xxEno	Enolase
26	xxFdp	Fructose-1,6-bisphosphatase I
27	xxGltA	Citrate synthase
28	xxIcd	Unphosphorylated isocitrate dehydrogenase
29	xxIcd-P	Phosphorylated isocitrate dehydrogenase
30	xxMaeAB	Malic enzymes MaeAB
31	xxMdh	Malate dehydrogenase
32	xxPckA	Phosphoenolpyruvate carboxykinase
33	xxPdh	Pyruvate dehydrogenase
34	xxPfkA	6-phosphofructokinase I
35	xxPpc	Phosphoenolpyruvate carboxylase
36	xxPpsA	Phosphoenolpyruvate synthase
37	xxPykF	Pyruvate kinase I
38	xxEIIA	Unphosphorylated PTS protein EIIA
39	xxEIIA-P	Phosphorylated PTS protein EIIA
40	xxEIICB	PTS protein EIICB
41	xxCra	Free Cra
42	xxCraFBP	Cra bound to fructose-1,6-bisphosphate
43	xxCrp	Free Crp
44	xxCrpcAMP	Crp bound to cyclic AMP
45	xxIclR	IclR
46	xxPdhR	free PdhR
46	xxPdhRPYR	PdhR bound to pyruvate

4 Generation of experimental data (simulated experiments)

Pseudo-experimental data was generated by simulation of the scenarios defined in the simulation files included as supplementary material in [1]. Simulated values of the model outputs, obtained at periodic time intervals, were taken as the experimental data for each scenario. The six scenarios and the corresponding data points (time instants) were:

1. Scenario 1: The population is adapted to glucose, the carbon source is glucose. 37 data points.
2. Scenario 2: The population is adapted to acetate, the carbon source is acetate. 8 data points.
3. Scenario 3: The population is adapted to glucose, the carbon source is acetate. 37 data points.
4. Scenario 4: The population is adapted to acetate, the carbon source is glucose. 51 data points.
5. Scenario 5: Diauxic shift scenario. 61 data points.
6. Scenario 6: Extended diauxic shift scenario (3 consecutive environments). 162 data points.

For optimization purposes, only the Scenario 6 was taken into account, that is, the objective function value was calculated using only the data points from Scenario 6. Data from the other scenarios was later used for validation purposes after model calibration, as shown in the next section.

5 Fits

Figures 1-6 show the plots of the fits between the calibrated model and the simulated experimental data for the 47 observables. Simulated measures are shown in blue and the model predictions are shown in red. Data corresponds to the six predefined scenarios described in the previous section.

In the plots, the x and y axes are not labeled for the sake of clarity. The x axis represents the time in hours, while the y axis represents the magnitude of each state; the units of the states are gl^{-1} for carbon sources, $\frac{\mu mol}{g_{DW}}$ for metabolites, $\frac{g_{Prot}}{g_{DW}}$ for proteins, and [OD] for the biomass concentration.

The pseudo-experimental data from Scenario 6 was used for calibration purposes. A nearly perfect fit was obtained, as can be seen in Figure 6. Then, we use data from Scenarios 1 to 5 for validation purposes. From Figures 1 to 5 it can be concluded that the calibrated model is capable of reproducing the system's behavior in these other scenarios reasonably well.

6 Influence of the τ parameter in the performance of CeSS

In order to see how the different options influence the algorithm's performance, we carry out optimizations using different intervals between information sharing: τ = 1.84, 2.14, 2.44, 2.62, and 2.84 (these values correspond to computation times of 1, 2, 3, and 5 days in the hardware used).

References

1. Kotte O, Zaugg J, Heinemann M: **Bacterial adaptation through distributed sensing of metabolic fluxes**. *Molecular Systems Biology* 2010, **6**(355).

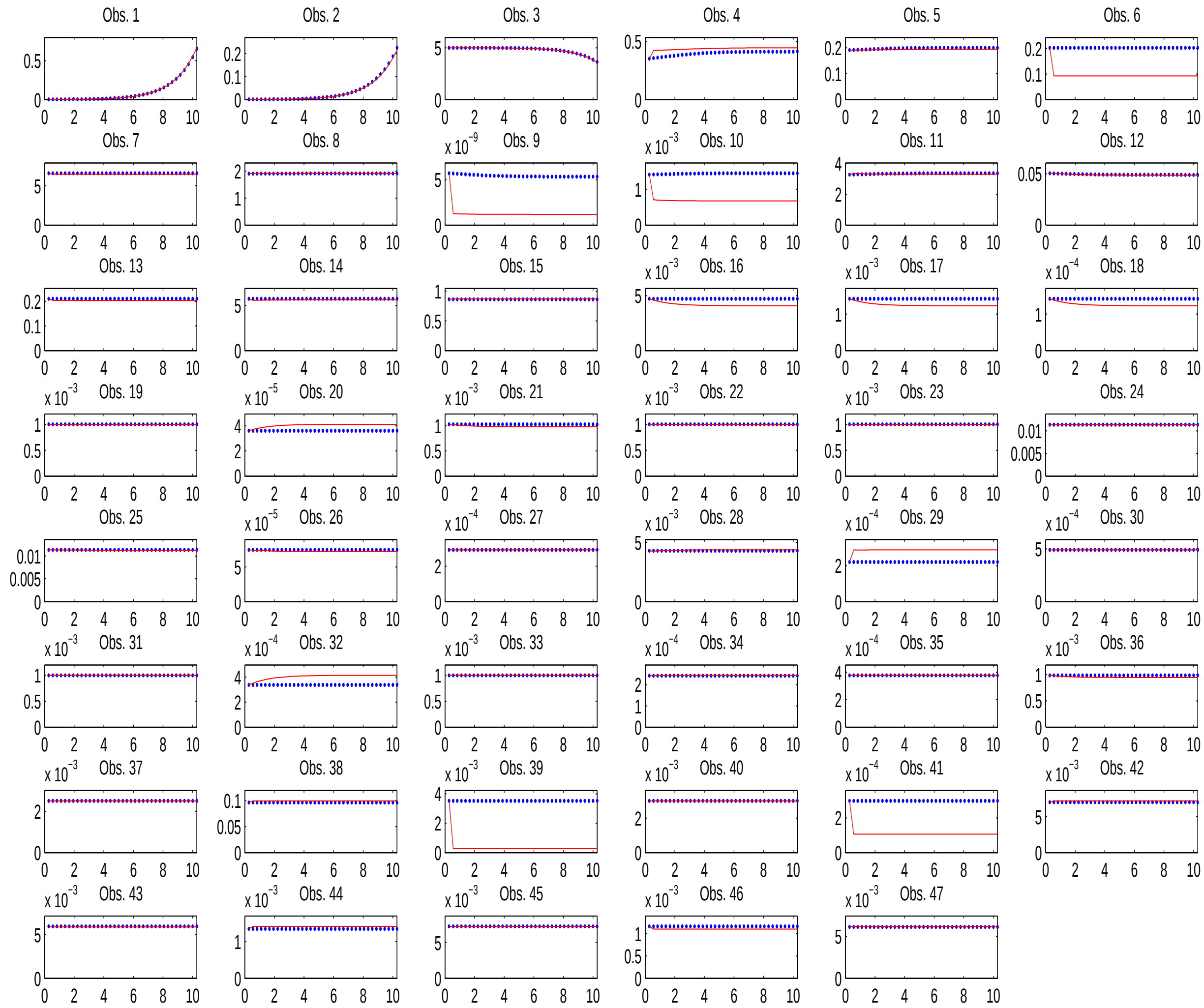

Figure 1: Model 2, Scenario 1. Pseudo-experimental data (blue points) vs. Calibrated model predictions (red line)

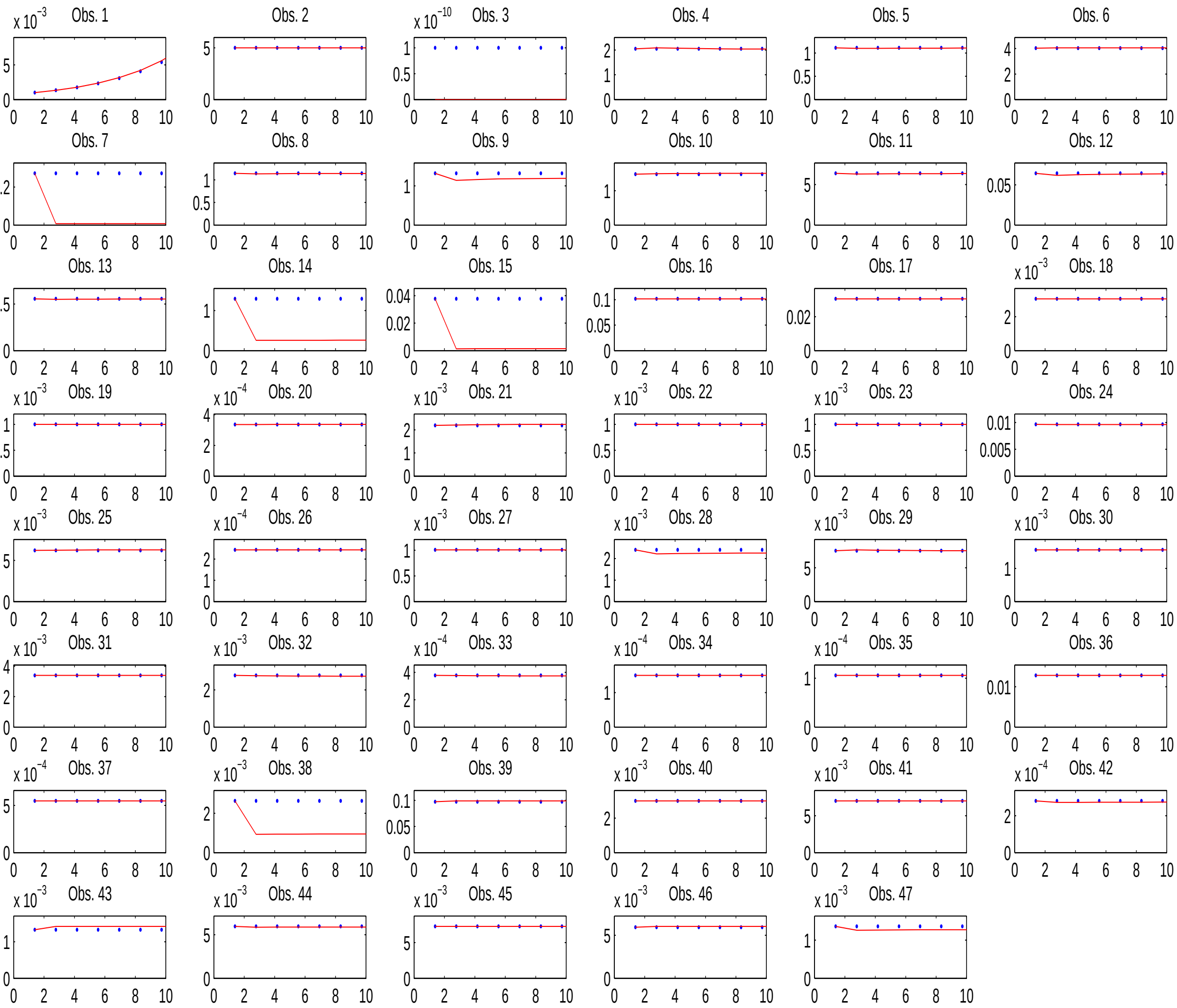

Figure 2: Model 2, Scenario 2. Pseudo-experimental data (blue points) vs. Calibrated model predictions (red line)

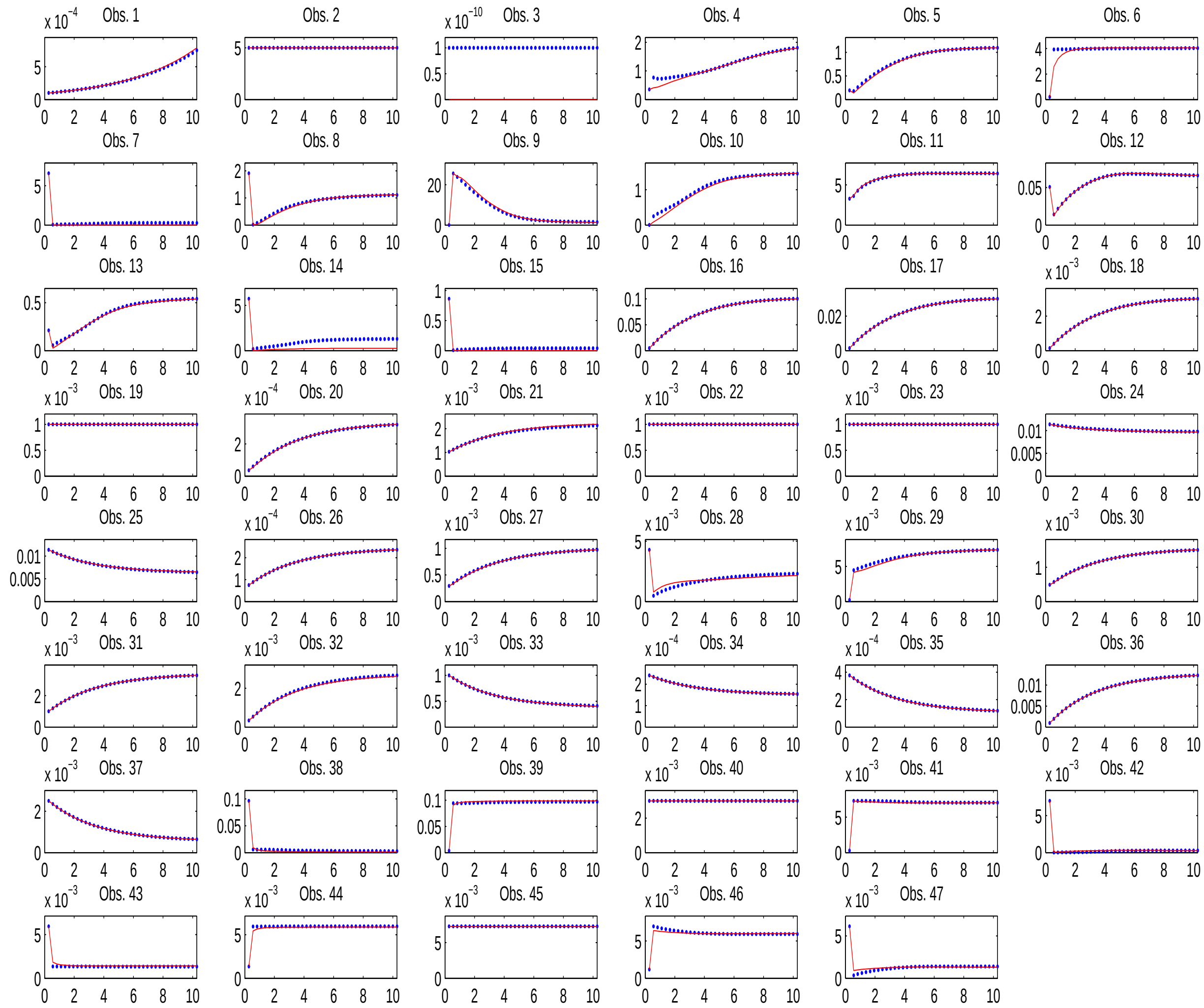

Figure 3: Model 2, Scenario 3. Pseudo-experimental data (blue points) vs. Calibrated model predictions (red line)

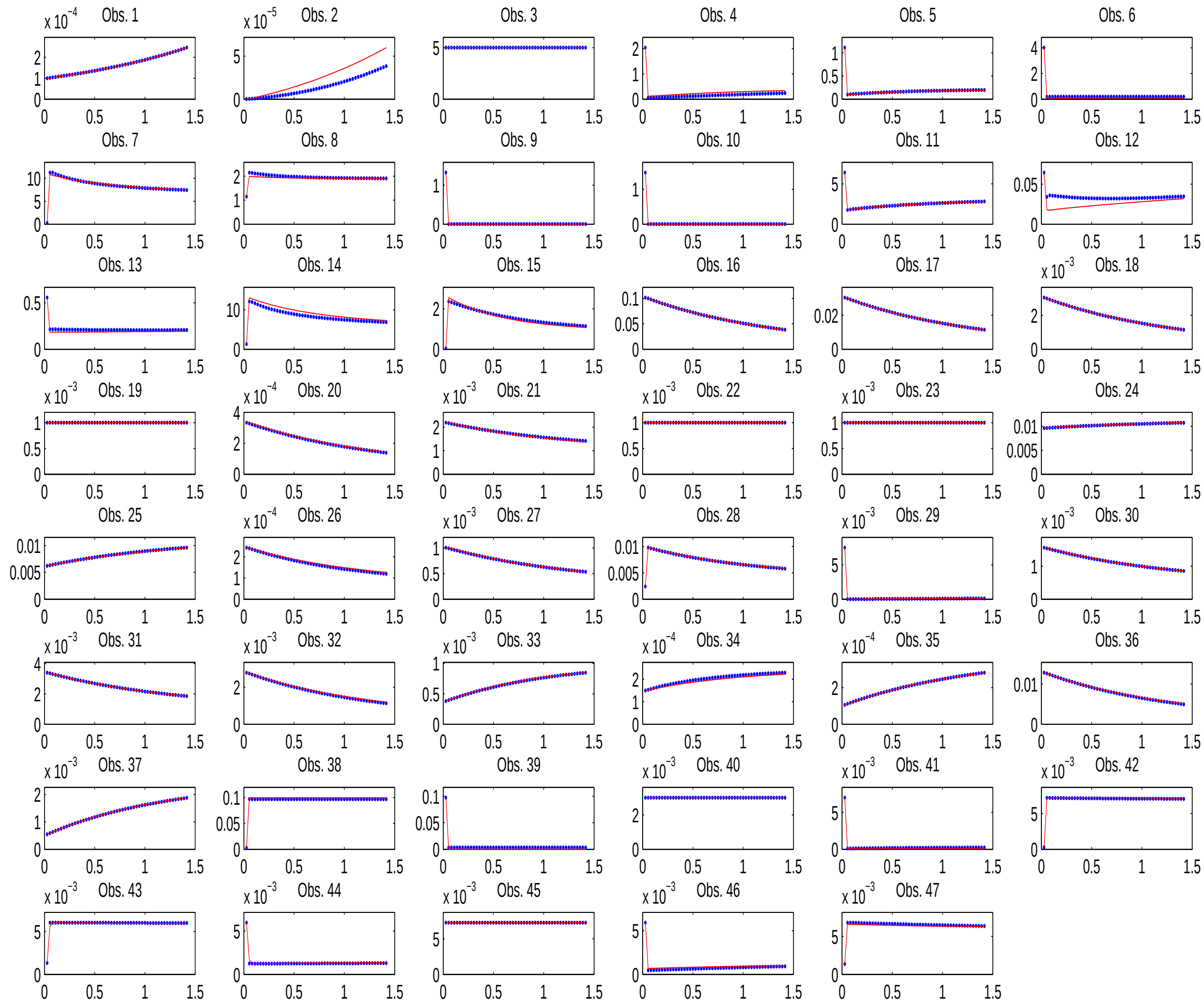

Figure 4: Model 2, Scenario 4. Pseudo-experimental data (blue points) vs. Calibrated model predictions (red line)

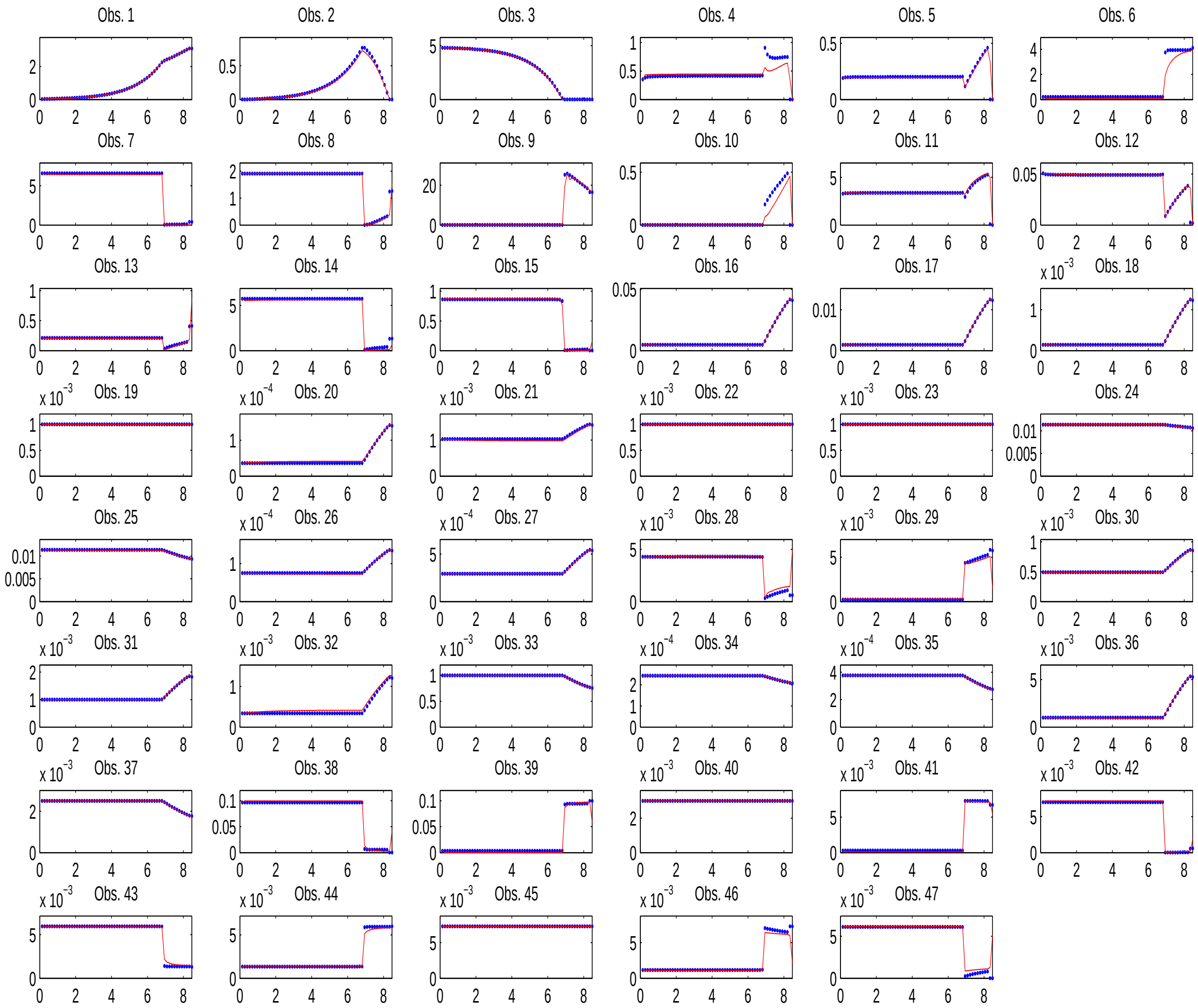

Figure 5: Model 2, Scenario 5. Pseudo-experimental data (blue points) vs. Calibrated model predictions (red line)

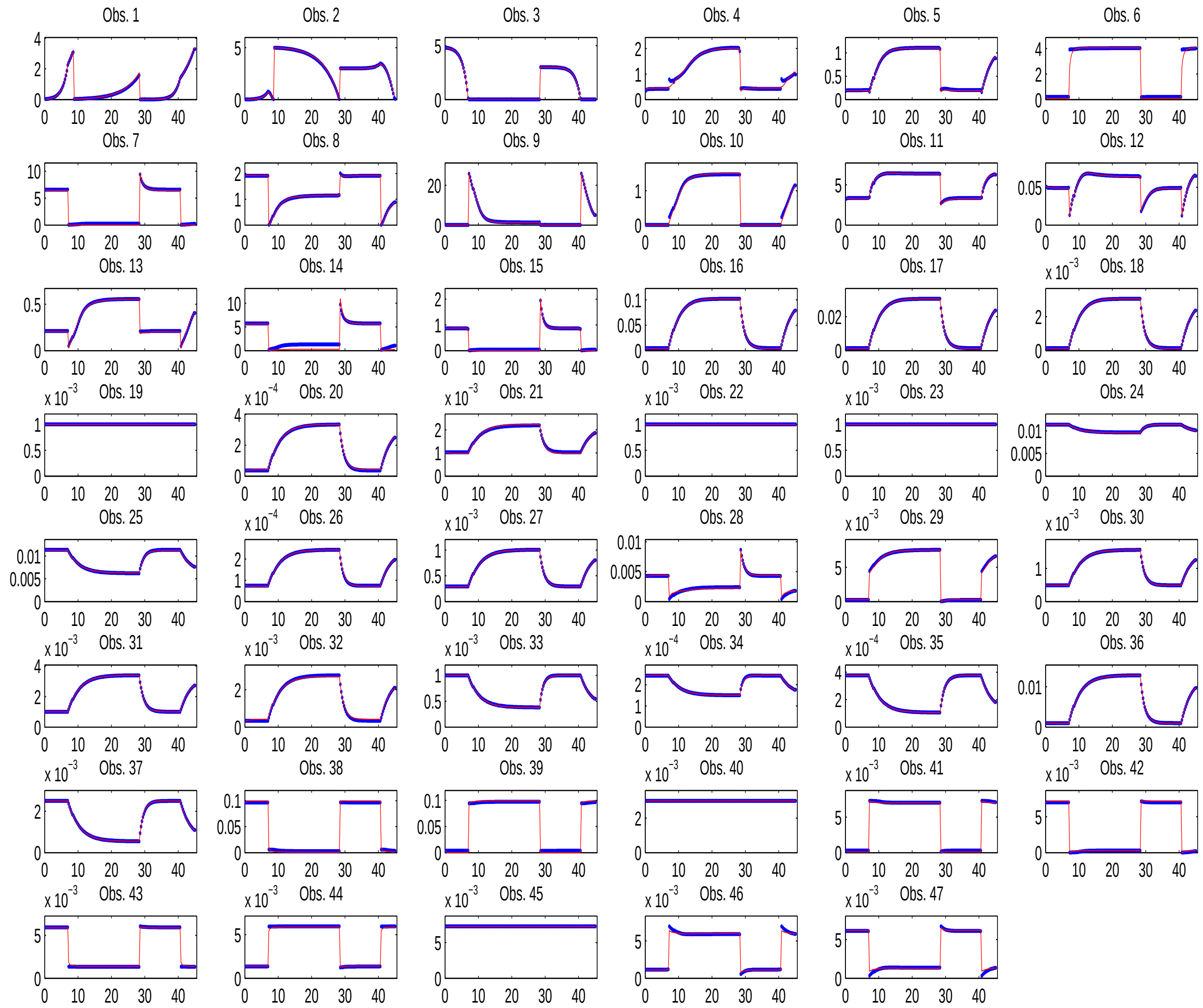

Figure 6: Model 2, Scenario 6. Pseudo-experimental data (blue points) vs. Calibrated model predictions (red line)

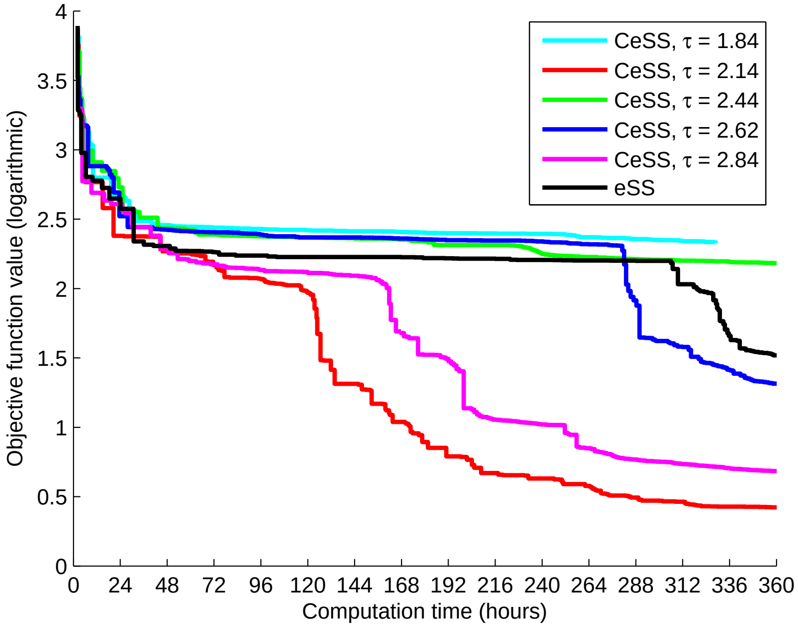

Figure 7: Convergence curves, different τ, Model 2.
